# Food consumption by degree of food processing and risk of type 2 diabetes mellitus: a prospective cohort analysis of the European Prospective Investigation into Cancer and Nutrition (EPIC)

**DOI:** 10.1016/j.lanepe.2024.101043

**Published:** 2024-09-16

**Authors:** Samuel J. Dicken, Christina C. Dahm, Daniel B. Ibsen, Anja Olsen, Anne Tjønneland, Mariem Louati-Hajji, Claire Cadeau, Chloé Marques, Matthias B. Schulze, Franziska Jannasch, Ivan Baldassari, Luca Manfredi, Maria Santucci de Magistris, Maria-Jose Sánchez, Carlota Castro-Espin, Daniel Rodríguez Palacios, Pilar Amiano, Marcela Guevara, Yvonne T. van der Schouw, Jolanda M.A. Boer, W.M. Monique Verschuren, Stephen J. Sharp, Nita G. Forouhi, Nicholas J. Wareham, Eszter P. Vamos, Kiara Chang, Paolo Vineis, Alicia K. Heath, Marc J. Gunter, Geneviève Nicolas, Elisabete Weiderpass, Inge Huybrechts, Rachel L. Batterham

**Affiliations:** a Centre for Obesity Research, Department of Medicine, University College London (UCL), London WC1E 6JF, UK; b Department of Public Health, Aarhus University, Aarhus, Denmark; c Steno Diabetes Center Aarhus, Aarhus University Hospital, Aarhus, Denmark; d Department of Nutrition, Sports and Exercise, University of Copenhagen, Frederiksberg, Denmark; e Danish Cancer Institute, Copenhagen, Denmark; f Department of Public Health, University of Copenhagen, Denmark; g Paris-Saclay University, UVSQ, Inserm, Gustave Roussy, CESP, Villejuif, France; h Department of Molecular Epidemiology, German Institute of Human Nutrition Potsdam-Rehbruecke, Nuthetal, Germany; i German Center for Diabetes Research (DZD), Neuherberg, Germany; j Institute of Nutritional Science, University of Potsdam, Nuthetal, Germany; k Department of Epidemiology and Data Science, Fondazione IRCCS Istituto Nazionale dei Tumori, Milan 20133, Italy; l Centre for Biostatistics, Epidemiology, and Public Health (C-BEPH), Department of Clinical and Biological Sciences, University of Turin, Regione Gonzole 10, Orbassano (TO), Italy; m A.O.U. Federico II, Naples, Italy; n Escuela Andaluza de Salud Pública (EASP), Granada 18011, Spain; o Instituto de Investigación Biosanitaria ibs.GRANADA, Granada 18012, Spain; p Centro de Investigación Biomédica en Red de Epidemiología y Salud Pública (CIBERESP), Madrid 28029, Spain; q Unit of Nutrition and Cancer, Catalan Institute of Oncology-ICO, L’Hospitalet de Llobregat, Barcelona, Spain; r Nutrition and Cancer Group, Epidemiology, Public Health, Cancer Prevention and Palliative Care Program, Bellvitge Biomedical Research Institute-IDIBELL, L’Hospitalet de Llobregat, Barcelona, Spain; s Department of Epidemiology, Murcia Regional Health Council-IMIB, Murcia, Spain; t CIBER Epidemiología y Salud Pública (CIBERESP), Madrid, Spain; u Ministry of Health of the Basque Government, Sub Directorate for Public Health and Addictions of Gipuzkoa, San Sebastian, Spain; v BioGipuzkoa (BioDonostia) Health Research Institute, Epidemiology of Chronic and Communicable Diseases Group, San Sebastián, Spain; w Instituto de Salud Pública y Laboral de Navarra, Pamplona 31003, Spain; x Centro de Investigación Biomédica en Red de Epidemiología y Salud Pública (CIBERESP), Madrid 28029, Spain; y Navarra Institute for Health Research (IdiSNA), Pamplona 31008, Spain; z Julius Center for Health Sciences and Primary Care, University Medical Center Utrecht, Utrecht University, Utrecht, the Netherlands; aa National Institute for Public Health and the Environment, Bilthoven, the Netherlands; ab Medical Research Council Epidemiology Unit, Institute of Metabolic Science, University of Cambridge Clinical School, Cambridge, UK; ac Public Health Policy Evaluation Unit, School of Public Health, Imperial College London, London W12 0BZ, UK; ad Department of Epidemiology and Biostatistics, School of Public Health, Imperial College London, London, UK; ae Nutrition and Metabolism Branch, International Agency for Research on Cancer, World Health Organization (IARC-WHO), Lyon, France; af International Agency for Research on Cancer, World Health Organization, Lyon, France; ag Bariatric Centre for Weight Management and Metabolic Surgery, University College London Hospital (UCLH), London NW1 2BU, UK; ah National Institute for Health Research, Biomedical Research Centre, University College London Hospital (UCLH), London W1T 7DN, UK

**Keywords:** Ultra-processed food, Food processing, Type 2 diabetes mellitus, Nova classification, Europe, Diet

## Abstract

**Background:**

It is unknown whether the association between ultra-processed food (UPF) intake and type 2 diabetes mellitus differs from other degrees of food processing. We examined the association between degree of food processing and incident type 2 diabetes mellitus.

**Methods:**

This was a prospective cohort analysis of the European Prospective Investigation into Cancer and Nutrition (EPIC). Dietary intake was assessed at baseline using dietary questionnaires and classified according to the Nova classification into unprocessed/minimally processed food (MPF), processed culinary ingredients (PCI), processed food (PF) and UPF. Type 2 diabetes mellitus cases were verified through multiple methods. Cox regression and statistical substitution analysis was used to estimate associations between MPF + PCI, PF and UPF intake and incident type 2 diabetes mellitus. To investigate heterogeneity in the association between UPF and incident type 2 diabetes mellitus, UPF sub-group analysis was conducted. Different reference groups were used in each analysis.

**Findings:**

Over an average 10.9 years follow-up of 311,892 individuals, 14,236 type 2 diabetes mellitus cases were identified. Each 10% increment of total daily food intake from UPF (%g/day) was associated with 17% (95% confidence interval (95%CI): 1.14–1.19) higher incident type 2 diabetes mellitus. Each 10% increment in MPF + PCI or PF intake was associated with lower incident type 2 diabetes mellitus (MPF + PCI hazard ratio: 0.94 (95%CI: 0.92–0.96); PF hazard ratio: 0.92 (95%CI: 0.89–0.95)). Replacing UPF with MPF + PCI or PF was associated with lower incident type 2 diabetes mellitus. However, heterogeneity was observed across UPF sub-groups, with breads, biscuits and breakfast cereals, sweets and desserts, and plant-based alternatives associated with lower incident type 2 diabetes mellitus.

**Interpretation:**

These findings support recommendations to focus on reducing intake of specific UPF for lowering type 2 diabetes mellitus risk.

**Funding:**

10.13039/100008700International Agency for Research on Cancer.

**Research in context**

**Evidence before this study**

PubMed was searched for peer-reviewed papers using: “ultra-processed food” AND “type 2 diabetes”, and: “Nova classification” AND “type 2 diabetes”, from conception until 28th May 2024 regarding prospective cohort associations between food processing according to the Nova classification and type 2 diabetes. Recent meta-analyses suggest increased risks of type 2 diabetes mellitus with greater UPF intake, with moderately convincing evidence for a dose–response association. However, less is known about whether the association between UPF intake and type 2 diabetes mellitus differs from other degrees of food processing.

**Added value of this study**

This is the first prospective study to examine the association between MPF + PCI, PF and UPF intake and incident type 2 diabetes mellitus. This study revealed contrasting associations between different degrees of food processing and incident type 2 diabetes mellitus, whereby replacing UPF with lower degrees of food processing was associated with lower incident type 2 diabetes mellitus. Importantly, this study identified that some UPF sub-groups were inversely associated with incident type 2 diabetes mellitus. Savoury snacks, animal-based products, ready-to-eat/heat mixed dishes and artificially- and sugar-sweetened beverages (ASB/SSB) were associated with higher incident type 2 diabetes mellitus, whereas breads, biscuits and breakfast cereals, sweets and desserts, and plant-based alternatives were associated with lower incident type 2 diabetes mellitus.

**Implications of all the available evidence**

The rising prevalence of type 2 diabetes mellitus is of concern across Europe, and worldwide. Our study provides important results that question the use of an overall UPF metric for public dietary guidance to reduce the risk of type 2 diabetes mellitus, and supports efforts to focus on reducing consumption of specific UPF.

## Introduction

Globally, 540 million individuals live with type 2 diabetes.^1^ The prevalence of type 2 diabetes mellitus has increased over four-fold in recent decades,^1^ and is expected to rise further.^2^ Individuals with type 2 diabetes mellitus have a lower quality of life,^3^ and increased risk of other cardiometabolic diseases and all-cause mortality.^1^ Modifiable risk factors for type 2 diabetes mellitus include adiposity, education, smoking, alcohol consumption, physical activity, sedentary behaviour, and diet.^4^^,^^5^ It is debated whether diets with a high proportion of ultra-processed food (UPF) pose health risks beyond the nutritional quality of the diet.^6^^,^^7^ The Nova classification, the most commonly used food processing classification, classifies foods into four categories.^8^ Category one are unprocessed/minimally processed foods (MPFs) which resemble the original intact food, such as whole fruits, vegetables, legumes, grains, meat and fish.^8^ Category two are processed culinary ingredients (PCIs), which are derived from MPFs, and added to such foods to make homemade dishes. PCIs include oil, sugar, and salt. Category three are processed foods (PFs), which combine MPFs and PCIs, such as tinned fish, salted or smoked meats, and fruits in syrup, or fruits, vegetables, or legumes in brine. Category four are UPF, and defined as industrial formulations using extracts of original foods, typically with many ingredients.^8^ UPFs tend to no longer resemble the original constituent food, and include processed meats, breakfast cereals, artificially- and sugar-sweetened beverages (ASB/SSB) and many ready-to-eat meals.

Globally, UPF intake is increasing,^9^^,^^10^ and constitutes a considerable proportion of dietary intake.^9^ Higher UPF intakes have been associated with adverse health outcomes, including weight gain and obesity,^11^ and cardiovascular disease.^12^ Studies also suggest increased risks of type 2 diabetes mellitus with greater UPF intake,^13^ with a meta-analysis of seven prospective cohort studies reporting a 37% higher risk of type 2 diabetes mellitus in the highest vs. lowest category of UPF intake.^14^ Another meta-analysis of prospective studies reported a 12% increased risk of type 2 diabetes mellitus with each 10% increment in UPF content of diets,^15^ with moderately convincing evidence for a dose–response association.^16^ An analysis across seven countries in the European Prospective Investigation into Cancer and Nutrition (EPIC) suggested UPF intake was associated with higher risk of cancer and cardiometabolic multimorbidity.^17^

These findings have led to calls for policy action on UPF. However, debate surrounds whether UPF intake should be reduced, or whether UPFs should be reformulated to improve their nutrient quality.^18^ Furthermore, there are several key questions that must be addressed to guide the most appropriate policy action. These include first, determining the association between all Nova groups and incident type 2 diabetes mellitus to identify whether UPF has the least favourable association, given that to date, all prospective studies except one have only examined the association of UPF with type 2 diabetes mellitus and not other Nova groups^19^; second, determining whether the associations are explained by current food- and nutrient-based dietary guidance, given the importance of distinguishing between UPFs and other foods high in fat, sugar and salt^20^; third, to quantify any potential benefit from replacing UPF intake with MPF, PCI or PF and therefore whether guidance to replace UPF with lower degrees of food processing is justified; fourth, to examine whether there is variation in the UPF association with incident type 2 diabetes mellitus across subgroups, to consider the value of an overall UPF metric; and fifth, understanding potential explanatory factors between UPF and incident type 2 diabetes mellitus.

## Methods

### Study design and participants

EPIC (epic.iarc.who.int) is a multi-centre, prospective cohort study. From 1992 to 2000, 521,323 participants were recruited from 23 centres across 10 European countries, including the United Kingdom (UK) (Oxford, Cambridge), France, Germany (Heidelberg, Potsdam), Greece, Italy (Varese, Turin, Florence, Naples, Ragusa), Denmark (Aarhus, Copenhagen), Norway, Spain (Asturias, Granada, Murcia, Navarra, San Sebastián-Gipuzkoa), Sweden (Malmø and Umeå) and The Netherlands (Bilthoven, Utrecht). In most cases, participants were selected from the general population. In the UK, 50% of the Oxford sample were non-meat eaters. Females only were recruited in France, Norway, Utrecht and Naples. State-school teachers were recruited in France, and members of blood donation associations (some Italian and Spanish centres), and females invited to breast cancer screening (Utrecht and Florence). Most participants were aged between 35 and 70 years at baseline.^21^

This analysis uses data from eight countries (Norway and Greece were excluded as incident type 2 diabetes mellitus cases were not ascertained). In total, 329,321 individuals were eligible for inclusion. Participants were then excluded if they had a follow-up length of zero (n = 935), had a baseline type 2 diabetes mellitus diagnosis (n = 9969), had implausible anthropometric measures (n = 227) as used in Cordova et al.^11^ (height <130 cm; body mass index (BMI) < 16.0 kg/m^2^; waist circumference (WC) > 160 cm; WC < 60 cm if BMI >25 kg/m^2^), or reported extreme energy intake to energy requirements (top and bottom 1%) (n = 6298)) (Supplementary Fig. S1).

### Ethics

The International Agency for Research on Cancer (IARC) and the Institutional Review Board at each centre approved the study. All participants provided written informed consent and were able to withdraw at any time. This current analysis was approved by the IARC Ethics Committee (IEC 23-04).

### Diet assessment

Dietary assessment was conducted at baseline using validated country- or centre-specific methods. Diet history questionnaires were used in France and Spain, semi-quantitative food frequency questionnaires (FFQs) were used in Italy (Northern Italy, Ragusa and Naples), the Netherlands and Denmark; and FFQs were used in the UK, Germany and Sweden. In Ragusa (Italy), Naples (Italy) and Spain, assessment was conducted via face-to-face interviews.^22^ In Spain and France, questionnaires were structured by meal. In Malmö (Sweden), a 7-day hot meal food record was also used. These methods have been validated within the EPIC source populations of interest.^22^ Data from questionnaires were combined following a standardised procedure (e.g., disaggregating local recipes and foods into ingredients) to generate a standardised food list and common food classification of >11,000 items that was comparable between countries.

### Nutrient intake

Responses to dietary assessments were used to estimate total daily food quantities (grams/day) at each centre.^21^ The EPIC nutrient database (ENDB) was then used to estimate nutrient intakes and to estimate total daily energy intake (kcal/day) for each participant.^23^ Whether salt was added to food was not assessed in most dietary questionnaires. Therefore, sodium in this analysis reflects the sodium content derived from food and does not include contributions from discretionary salt (and therefore not total sodium intake). Nutrients consumed from dietary supplements were also not included.

### Diet quality indices

Several diet quality indices were used to adjust associations between Nova groups and type 2 diabetes mellitus, including a Mediterranean diet score,^24^ the Food Standards Agency Nutrient Profiling Model Dietary Index (i.e., NutriScore^25^), and the inflammatory score of the diet.^26^ Adherence to the UK Eatwell Guide (EWG) was constructed using a previously published metric that dichotomised adherence (yes, no) for nine recommendations (fruit and vegetables, total fat, saturated fat, sugar, salt, fibre, red and processed meat, oily fish, and other fish)^27^ (see Supplementary materials and Table S1). These diet quality indices were chosen as they reflect European public health dietary guidance (NutriScore and EWG), indices to healthy diets (Mediterranean diet), and factors proposed to drive effects of UPF (inflammatory score).

### Nova classification

Full details on the coding of dietary assessments into Nova is available elsewhere.^22^ In brief, foods and drinks were coded as MPF if they were unprocessed or modified through methods such as drying, boiling, or freezing, and did not include salt, added sugar, oils or other substances. Foods were coded as PCI if they were directly obtained from MPFs or nature (e.g., table sugar, olive oil, butter, and salt). To ensure accurate estimation of PCI, homemade dishes were first disaggregated using local recipes, with coding into Nova applied to the constituent ingredients. Items were coded as PF if they were simple industrial combinations of MPFs and PCIs, potentially using basic preservation methods such as canning or bottling. Items were coded as UPF if they were largely made from formulations of industrially modified ingredients, and contained additives such as colours, flavours, emulsifiers or gelling agents. Intake of foods (grams/day) in each Nova group were summed for each participant. Further details of the definition are in the Supplementary materials.

The main exposure variables were: 1) MPF + PCI, 2) PF, and 3) UPF, as percentage weight of total food intake (%g/day), to better capture non-nutritive aspects of UPF as the exposure variable. PCIs were combined with MPFs (MPF + PCI) as they are typically consumed alongside MPFs in culinary preparations, and in EPIC, home-made dishes were disaggregated into constituent ingredients (i.e., MPF and PCI) to better estimate PCI intake.

Upper-, middle- and lower-bound scenario estimates of Nova group intake were constructed to consider the potential changes in industrial food processing since the 1990s.^22^ The lower- and upper-bound estimates represent scenarios with the lowest and highest degrees of processing, respectively. In these scenarios, a small number of ambiguous items were recoded based on lower or higher degrees of processing. The main analysis was conducted using the middle-bound scenario for all Nova groups, which has been previously validated.^22^ Further detail is provided elsewhere.^22^

### Covariates

General data collection on a range of sociodemographic and health variables was conducted at recruitment using validated questionnaires. Covariates in this analysis include study centre, self-reported sex (male, female), highest education level (none, primary school, technical/professional, secondary, higher education, not specified/missing), occupation (employed, housewife, retired, unemployed, student, other, missing), history of previous illness (cardiovascular disease, cancer, hypertension, hyperlipidaemia), family history of type 2 diabetes mellitus in a parent or sibling (yes, no, missing), smoking status and intensity (never, current 1–15 cigarettes/day, current 16–25 cigarettes/day, current 26+ cigarettes/day, quit £10 years, quit 11–20 years, quit >20 years, current pipe/cigar/occasional smoker, current/former with missing intensity, missing), physical activity level (inactive, moderately inactive, moderately active, active, missing), alcohol intake (g/day), menopausal status (premenopausal, postmenopausal, perimenopausal, surgical postmenopausal), and use of hormone replacement therapy (HRT) or oral contraceptives (yes, no). Anthropometrics (weight, height, and WC) were measured at each centre, except in France and Oxford (UK), where values were self-reported. These self-reported measures have been shown to be valid for epidemiological analyses.^28^ Body mass index (BMI) was calculated from weight and height measurements as weight (kg)/height squared (m^2^), and waist-height-ratio (WHtR) was calculated from waist and height measurements as waist (cm)/height (cm), and Relative Fat Mass (RFM) as (20∗height (m)/waist (m)) + (12∗sex), where sex = 0 (males), and sex = 1 (females).^29^ Missing data for highest education level (2%), family history of type 2 diabetes mellitus (50%), occupation (55%), smoking intensity (1%) and physical activity level (9%) were coded as a missing category.

### Outcome assessment

Case ascertainment for type 2 diabetes mellitus in EPIC has been described previously.^30^ Briefly, incident type 2 diabetes mellitus was obtained from a range of sources at each centre, including self-report, primary- and secondary-care register linkage, medications registers, hospital admissions and mortality data.^30^ Cases in Denmark and Sweden were obtained from local and national diabetes and pharmaceutical registers. In other countries, further sources of verification of type 2 diabetes mellitus status were used when only one data source was available, including evidence of diabetes medication, hospital admissions data, diabetes code on death certificate, review of individual medical records at some centres.^30^ In the Netherlands, cases were validated by contacting the general practitioner for confirmation using a set of questions.

### Statistical analyses

#### Aim 1

The association between the three exposure variables and incident type 2 diabetes mellitus was assessed using Cox proportional hazards regression with age as the underlying time variable. Time at entry was age at recruitment, and exit time was age at type 2 diabetes mellitus diagnosis, end of follow-up, loss to follow-up, or death, whichever came first. Hazard ratios (HRs) with 95% confidence intervals (95%CI) were calculated for a continuous 10% g/day increase in intake, and across sex-specific quartiles. The adjustment set was identified by constructing a directed acyclic graph (DAG) (Supplementary Fig. S2). Model 1 was unadjusted, with either MPF + PCI, PF or UPF as the independent variable. Model 2 was adjusted for sociodemographics (sex, study centre) socioeconomic status (education level, occupation), family history of type 2 diabetes mellitus, lifestyle behaviours (smoking status and intensity, alcohol intake, physical activity) and total energy intake (i.e., replacing a 10% increment in weight from one Nova group, with a corresponding lower weighted average of other groups not included in the model, whilst holding energy intake constant).

To investigate the association between food processing and incident type 2 diabetes mellitus independent of dietary nutrient content, model 2 adjusted for saturated fat, sugar, and sodium intake (model 3). To account for diet quality, model 2 adjusted for Mediterranean diet adherence (model 4). Model 5 was model 2 adjusted for the intake of saturated fat, sugar and sodium, and Mediterranean diet adherence. Models 2 and 5 were also adjusted for WHtR, and height (as a type 2 diabetes mellitus risk factor^31^) (separately adjusting for each).

#### Aim 2

As the percentage of dietary intake from all Nova groups represents compositional data, statistical substitution modelling was performed to consider the association of replacing 10% g/day from MPF + PCI, PF or UPF for another food processing group(s) on incident type 2 diabetes mellitus. The ‘leave-one-out’ method was used. Either MPF + PCI, PF or UPF was left out from each model (e.g., in a model including PF and UPF, the HR for UPF represents the substitution effect of replacing 10% g/day of MPF + PCI with UPF, whilst keeping PF intake constant). Nova variables were adjusted for covariates as per model 2, and model 5 to consider the substitution effect independent of dietary quality and nutrient profile. Total energy intake was adjusted for, to account for confounding influences of body size and dietary misreporting.^32^

#### Aim 3

To assess heterogeneity in the association between UPF and incident type 2 diabetes mellitus, the association between each UPF sub-group and incident type 2 diabetes mellitus was investigated. UPF sub-groups were simultaneously entered into model 2 without the overall UPF variable, to examine the association between a 10% g/day increase in each UPF sub-group and incident type 2 diabetes mellitus adjusted for other UPF sub-groups (see Supplementary Table S2 for UPF sub-groups).

#### Aim 4

To understand potential explanatory factors for the association between UPF and incident type 2 diabetes mellitus, mediation analysis was conducted for the adiposity indicator, WHtR. A regression-based mediation analysis was used to determine the pure natural direct effect (PNDE) (the effect on the outcome from a one unit increase in the exposure, holding the mediator constant at the level in the absence of the exposure, i.e., the effect of UPF intake on incident type 2 diabetes mellitus), total natural indirect effect (TNIE) (effect on the outcome from a one unit increase in the mediator, holding the exposure constant at the level in the presence of the exposure, i.e., the effect of UPF intake on incident type 2 diabetes mellitus, via the mediator) of WHtR.^33^ Closed-form parameter function estimation was used, with delta-method standard errors, 95% CIs and p-values. Mediation analyses were conducted using model 2 covariates. First, linear regressions were constructed between each Nova group and the potential mediator. Then, Cox models were constructed, including the mediating variable. Total effects (TE) (the sum of the PNDE and TNIE) were then decomposed into the direct and indirect effect to estimate the proportion mediated (PM) (the ratio of TNIE to TE). The analysis made strong assumptions of no unmeasured mediator-outcome confounding.^33^

To test the proportional hazards assumption for the main analysis, Schoenfeld residuals were generated for the adjusted Cox models (model 2) (p-values: MPF + PCI = 0.236; PF = 0.734; UPF = 0.068). To further confirm whether the proportional hazards assumption was met, Nova groups were modelled as a time-varying covariate (i.e., whether the HR varies with time). Flexible parametric models were constructed on the cumulative hazard scale, with restricted cubic splines with 5 internal knots to model the baseline hazard function. Age was used as the time scale, with the Nova variable modelled as a time-varying coefficient with 2 knots. HRs for a 10% g/day increase in intake were plotted against age at follow-up to determine the appropriateness of a summary HR over the follow-up duration. This was deemed suitable for the majority (>90%) of the sample (see Supplementary materials and Fig. S3).

Non-linearity was assessed by modelling each Nova variable using restricted cubic splines. Adjusted for model 2 covariates, knots were placed at the 10th, 50th and 90th percentiles, and the relative hazard was then plotted against the respective Nova variable (%g/day).

### Sensitivity analyses

Sensitivity analyses were performed with models 2 and 5 for MPF + PCI, PF and UPF, including with different covariates to assess the stability and confidence in results and to further examine explanatory dietary factors, and with alternate exposure variables (see Supplementary materials). Models were repeated excluding the first two years of follow-up, excluding participants with CVD or hypertension at baseline, and using complete cases only. To assess potential heterogeneity, interaction terms were added between UPF intake and sex, country, BMI and Mediterranean diet adherence. For statistically significant interactions, models were repeated within subgroups. Lastly, E-values were calculated for the main analyses, to identify the minimum strength of association with the exposure and outcome that an unmeasured confounder would need, in order to explain away the exposure-outcome association, conditional on measured covariates.^34^

Analyses were conducted in R, version 2023.09.1 + 494 and Stata, version 18.0. In R, Cox models were computed using “survival”, restricted cubic splines using “rms”, mediation analyses using “CMAverse”,^35^ and DAG using “dagitty”.^36^ In Stata, Schoenfeld residuals were assessed using “estat”, and flexible parametric survival models were constructed using “stpm2”. Statistical significance was set at p < 0.05.

### Patient and public involvement

Patients and the public were not involved in the design and conduct of this analysis, as the data used were pseudo-anonymised and participants were not contactable.

### Role of funding source

The study funders had no role in the design, analysis, interpretation, or writing of the manuscript.

## Results

Overall, 311,892 participants were included in the analysis. Included vs. excluded participants differed slightly, including amongst others, a younger age and higher education level (Supplementary Table S3). At recruitment, average age was 52.5 years (standard deviation (SD): 9.4), BMI was 25.7 kg/m^2^ (SD: 4.1), and 63.5% of the participants were female. Average follow-up time was 10.9 years (SD: 2.4), during which 14,236 (4.6%) participants were diagnosed with type 2 diabetes mellitus. The average percentage contribution of UPF to total daily dietary intake in grams was 13.0% (SD: 7.8). MPF + PCIs contributed 72.1% (SD:12.1) (MPF: 70.8% (SD: 12.3), PCI: 1.3% (SD: 1.1)) and PFs 14.9% (SD: 10.6) (Table 1 and Supplementary Fig. S4). Nova group contributions to dietary intakes by country are shown in Supplementary Fig. S5 and Table S4. The UK had the highest contribution of UPF to daily intake (17.4% (SD: 8.0)), and France the lowest (6.9% (SD: 4.2)). France had the highest contribution of MPF + PCI to daily intake (81.4% (SD: 7.5)), and Italy the lowest (63.8% (SD: 11.0)). Italy had the highest contribution of PF to daily intake (25.7% (SD: 10.4)), and the UK the lowest (7.6% (SD: 6.4)).

**Table 1:** Characteristics of participants across sex-specific quartiles of UPF intake.

Variable	All participants (311,892)	Quartile 1 (77,974)	Quartile 2 (77,972)	Quartile 3 (77,972)	Quartile 4 (77,974)	p-value
%g/day						
MPF + PCI	72.1 (12.1)	76.4 (13.0)	75.3 (10.9)	72.5 (9.8)	64.2 (10.6)	<0.001
MPF	70.8 (12.3)	74.8 (13.3)	74.1 (11.3)	71.4 (10.2)	63.2 (10.8)	<0.001
PCI	1.3 (1.1)	1.7 (1.2)	1.2 (1.1)	1.1 (1.0)	1.0 (1.0)	<0.001
PF	14.9 (10.6)	18.4 (12.8)	15.2 (10.6)	13.7 (9.4)	12.3 (8.2)	<0.001
UPF	13.0 (7.8)	5.1 (1.9)	9.5 (1.5)	13.7 (1.8)	23.5 (7.2)	<0.001
%kcal/day						
MPF + PCI	42.8 (12.5)	51.9 (12.6)	44.0 (10.8)	40.1 (10.0)	35.1 (9.8)	<0.001
MPF	35.0 (10.4)	40.8 (10.7)	36.4 (9.5)	33.5 (9.0)	29.4 (8.8)	<0.001
PCI	7.8 (6.3)	11.1 (6.8)	7.6 (6.2)	6.6 (5.6)	5.8 (5.2)	<0.001
PF	26.2 (11.3)	30.9 (11.9)	26.7 (10.8)	24.6 (10.4)	22.4 (10.2)	<0.001
UPF	31.0 (14.4)	17.2 (10.3)	29.2 (10.8)	35.3 (10.9)	42.5 (11.9)	<0.001
Age (at baseline in years) (SD)	52.5 (9.4)	53.3 (8.0)	53.3 (8.6)	52.5 (9.6)	50.8 (10.7)	<0.001
Sex						1.000
Men	113,746 (36%)	28,437 (36%)	28,436 (36%)	28,436 (36%)	28,437 (36%)	
Women	198,146 (64%)	49,537 (64%)	49,536 (64%)	49,536 (64%)	49,537 (64%)	
Highest education level						<0.001
None	12,606 (4%)	7136 (9%)	2363 (3%)	1726 (2%)	1381 (2%)	
Primary school	92,900 (30%)	25,565 (33%)	22,932 (29%)	22,019 (28%)	22,384 (29%)	
Technical/professional school	78,809 (25%)	12,907 (17%)	19,556 (25%)	22,106 (28%)	24,240 (31%)	
Secondary school	51,827 (17%)	14,252 (18%)	13,442 (17%)	12,695 (16%)	11,438 (15%)	
Longer education (incl. University degree)	68,741 (22%)	17,188 (22%)	18,470 (24%)	17,457 (22%)	15,626 (20%)	
Not specified/Missing	7009 (2%)	926 (1%)	1209 (2%)	1969 (3%)	2905 (4%)	
Current occupation						<0.001
Employed	94,764 (30%)	10,677 (14%)	21,866 (28%)	28,118 (36%)	34,103 (44%)	
Housewife	10,553 (3%)	871 (1%)	2103 (3%)	3363 (4%)	4216 (5%)	
Retired	25,546 (8%)	3649 (5%)	6181 (8%)	7618 (10%)	8098 (10%)	
Unemployed	6551 (2%)	812 (1%)	1374 (2%)	1774 (2%)	2591 (3%)	
Student	1023 (0.3%)	112 (0.1%)	206 (0.3%)	319 (0.4%)	386 (0.5%)	
Other	2527 (1%)	194 (0.2%)	450 (1%)	712 (1%)	1171 (2%)	
Missing	170,928 (55%)	61,659 (79%)	45,792 (59%)	36,068 (46%)	27,409 (35%)	
Height (cm)	166.8 (9.3)	164.9 (9.1)	167.1 (9.3)	167.5 (9.2)	167.6 (9.3)	<0.001
BMI (kg/m2)	25.7 (4.1)	26.0 (4.1)	25.6 (4.0)	25.5 (4.0)	25.7 (4.2)	<0.001
WC (cm)	85.4 (12.5)	86.3 (12.6)	85.1 (12.3)	84.9 (12.3)	85.1 (12.8)	<0.001
WHtR	0.5 (0.1)	0.5 (0.1)	0.5 (0.1)	0.5 (0.1)	0.5 (0.1)	<0.001
RFM	32.0 (6.5)	33.2 (6.5)	31.9 (6.5)	31.4 (6.3)	31.6 (6.4)	<0.001
Smoking status						<0.001
Never	144,105 (46%)	35,797 (46%)	35,071 (45%)	36,076 (46%)	37,161 (48%)	
Former	88,481 (28%)	21,215 (27%)	22,874 (29%)	22,931 (29%)	21,461 (28%)	
Current	75,877 (24%)	20,108 (26%)	19,274 (25%)	18,130 (23%)	18,365 (24%)	
Missing	3429 (1%)	854 (1%)	753 (1%)	835 (1%)	987 (1%)	
Smoking status and intensity						<0.001
Never	130,582 (42%)	30,550 (39%)	31,382 (40%)	33,304 (43%)	35,346 (45%)	
Current, 1–15 cigarettes/day	38,770 (12%)	9232 (12%)	10,044 (13%)	9632 (12%)	9862 (13%)	
Current, 16–25 cigarettes/day	21,127 (7%)	5623 (7%)	5273 (7%)	4964 (6%)	5267 (7%)	
Current, 26+ cigarettes/day	5248 (2%)	1735 (2%)	1225 (2%)	1058 (1%)	1230 (2%)	
Former, quit ≤ 10 years	30,818 (10%)	8104 (10%)	7759 (10%)	7626 (10%)	7329 (9%)	
Former, quit 11–20 years	27,539 (9%)	6785 (9%)	7009 (9%)	7072 (9%)	6673 (9%)	
Former, quit 20+ years	26,697 (9%)	5735 (7%)	7178 (9%)	7279 (9%)	6505 (8%)	
Current, pipe/cigar/occas	23,089 (7%)	8786 (11%)	6135 (8%)	4789 (6%)	3379 (4%)	
Current/Former, missing	5086 (2%)	861 (1%)	1344 (2%)	1467 (2%)	1414 (2%)	
Missing	2936 (1%)	563 (1%)	623 (1%)	781 (1%)	969 (1%)	
Physical activity level						<0.001
Inactive	53,197 (17%)	14,085 (18%)	14,200 (18%)	12,945 (17%)	11,967 (15%)	
Moderately inactive	86,808 (28%)	24,177 (31%)	21,956 (28%)	20,197 (26%)	20,478 (26%)	
Moderately active	116,250 (37%)	30,665 (39%)	27,411 (35%)	28,563 (37%)	29,611 (38%)	
Active	28,236 (9%)	6037 (8%)	6665 (9%)	7361 (9%)	8173 (10%)	
Missing	27,401 (9%)	3010 (4%)	7740 (10%)	8906 (11%)	7745 (10%)	
Family history of type 2 diabetes mellitus (in parent or sibling)						<0.001
No	127,751 (41%)	24,435 (31%)	34,829 (45%)	35,993 (46%)	32,494 (42%)	
Yes	28,271 (9%)	4844 (6%)	7791 (10%)	8298 (11%)	7338 (9%)	
Missing	155,870 (50%)	48,695 (62%)	35,352 (45%)	33,681 (43%)	38,142 (49%)	
Baseline cardiovascular disease, cancer, hypertension, hyperlipidaemia						<0.001
No (for all)	103,512 (33%)	35,737 (46%)	24,833 (32%)	21,197 (27%)	21,745 (28%)	
Yes (for at least one)	100,430 (32%)	27,048 (35%)	24,237 (31%)	24,122 (31%)	25,023 (32%)	
Do Not Know (for at least one)	22,407 (7%)	3757 (5%)	6776 (9%)	6108 (8%)	5766 (7%)	
Missing (none of above)	85,543 (27%)	11,432 (15%)	22,126 (28%)	26,545 (34%)	25,440 (33%)	
Energy intake (kcal/day)	2121.8 (604.1)	2038.1 (602.2)	2069.7 (579.3)	2135.4 (584.8)	2244.0 (628.3)	<0.001
Energy Density (kcal/100 g)	83.1 (28.3)	84.5 (32.7)	79.7 (29.0)	82.1 (25.7)	86.1 (24.7)	<0.001
Diet weight (g/day)	2758.9 (958.7)	2703.7 (1078.2)	2820.8 (969.2)	2760.0 (876.1)	2751.0 (894.2)	<0.001
Alcohol (g/day)	13.3 (17.9)	16.7 (22.3)	14.2 (18.1)	12.2 (15.6)	10.0 (14.0)	<0.001
Protein (g/day)	88.1 (26.9)	90.4 (28.5)	87.3 (26.5)	87.5 (26.0)	87.3 (26.2)	<0.001
Carbohydrate (g/day)	234.4 (73.5)	217.9 (72.4)	225.3 (67.9)	236.1 (68.6)	258.4 (78.2)	<0.001
Sugar (g/day)	105.4 (43.6)	89.3 (34.9)	96.7 (36.1)	107.0 (39.4)	128.6 (51.7)	<0.001
Fibre (g/day)	22.7 (7.6)	23.2 (7.9)	22.6 (7.4)	22.6 (7.4)	22.5 (7.6)	<0.001
Fat (g/day)	82.0 (28.6)	76.4 (26.7)	80.0 (27.2)	84.0 (28.4)	87.9 (30.6)	<0.001
Saturated Fat (g/day)	32.0 (12.7)	27.5 (11.2)	31.5 (11.9)	33.6 (12.6)	35.3 (13.6)	<0.001
Monounsaturated fat (g/day)	30.3 (12.3)	30.7 (13.5)	29.4 (12.1)	30.0 (11.7)	30.9 (11.9)	<0.001
Polyunsaturated fat (g/day)	13.2 (5.8)	12.0 (5.7)	12.5 (5.2)	13.5 (5.6)	14.6 (6.3)	<0.001
Sodium (mg/day)	2748.9 (1052.7)	2574.0 (988.1)	2755.3 (1059.5)	2814.0 (1069.5)	2852.5 (1069.7)	<0.001
Mediterranean Diet Adherence	8.0 (3.1)	9.6 (3.1)	8.1 (3.0)	7.5 (2.9)	6.9 (2.8)	<0.001
NutriScore	5.9 (2.1)	4.8 (2.0)	5.8 (1.8)	6.3 (1.8)	7.0 (2.0)	<0.001
Eatwell Guide Adherence (1–9)	3.2 (1.6)	4.1 (1.6)	3.3 (1.5)	2.9 (1.5)	2.6 (1.5)	<0.001
Inflammatory Score of the Diet	0.6 (1.7)	0.6 (1.7)	0.7 (1.6)	0.6 (1.7)	0.6 (1.7)	<0.001

BMI: body mass index; MPF: unprocessed/minimally processed food; PCI: processed culinary ingredients; PF: processed food; RFM: relative fat mass; SD: standard deviation; type 2 diabetes mellitus; UPF: ultra-processed food; WC: waist circumference; WHtR: waist-to-height ratio.

Participants in the highest vs. lowest quartile of UPF intake were more likely to be younger, currently employed, and less likely to have no baseline disease. Participants in the highest vs. lowest quartile also had higher intake of energy, total fat, saturated fat, polyunsaturated fat, carbohydrate, sugar and sodium, and lower intake of alcohol, and protein, lower adherence to the EWG and Mediterranean diet, and a higher dietary energy density and Nutri-Score. Participant characteristics across sex-specific quartiles of MPF + PCI and PF intake are provided in Supplementary Table S5.

Dietary contributions by food sub-group across Nova groups are shown in Supplementary Table S6, and contributions by UPF sub-group across sex-specific quartiles of RFM in Supplementary Table S7. Tea, coffee, water, fruit, vegetables, milk and plain yoghurt were the main contributors to MPF intake. Table sugar, plant oil and animal fat were the main contributors to PCI intake. Bread, beer and wine and cheese were the main contributors to PF intake. ASB/SSB, breads, biscuits and breakfast cereals, sweets and desserts, animal-based products and ready-to-eat/heat mixed dishes were the main contributors to UPF intake.

### Aim 1

**Fig. 1:**
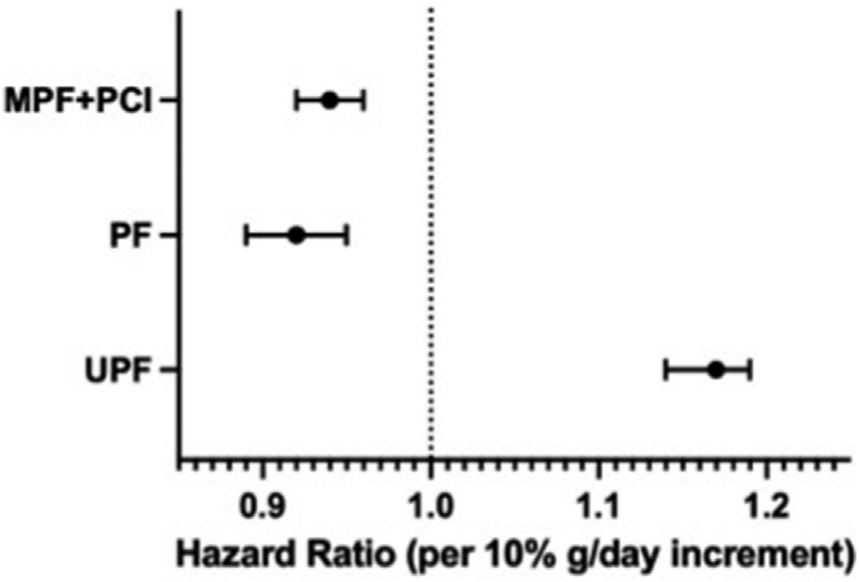
Hazard ratios and 95% confidence intervals of the association between Nova group intake and incident type 2 diabetes mellitus. Associations between Nova group intake and incident type 2 diabetes mellitus in EPIC. Error bars indicate 95% confidence intervals. Nova group intakes expressed as a percentage of daily dietary intake (%g/day). Hazard ratios expressed per 10%g/day increase in Nova group intake, in three separate models. Cox proportional hazard models, with age as the underlying time variable. Time at entry was age at recruitment, and exit time was age at type 2 diabetes mellitus diagnosis, end of follow-up, loss to follow-up, or death, whichever came first. Models were adjusted for sex, occupation, study centre, education level, smoking status and intensity, physical activity level, alcohol intake, family history of diabetes and total energy intake. Abbreviations: MPF: unprocessed/minimally processed food; PCI: processed culinary ingredients; PF: processed food; UPF: ultra-processed food.

Associations between Nova group intakes and incident type 2 diabetes mellitus are shown in Table 2 and Fig. 1. Visual inspection of restricted cubic spline plots indicated linearity across MPF + PCI until high intakes (reverse J-shape), some non-linearity (U shape) across PF intake, and linearity across UPF intake (Supplementary Fig. S6). In model 2, higher UPF intake was associated with higher incident type 2 diabetes mellitus (HR per 10% g/day: 1.17 (95%CI: 1.14–1.19)). Higher MPF + PCI and PF intake were associated with lower incident type 2 diabetes mellitus (MPF + PCI HR per 10% g/day: 0.94 (95%CI: 0.92–0.96); PF HR per 10% g/day: 0.92 (95%CI: 0.89–0.95)). Results were similar after further adjusting model 2 for saturated fat, sugar and sodium intake (model 3), for Mediterranean diet adherence (model 4), and for saturated fat, sugar, sodium and Mediterranean diet adherence (model 5). The associations between MPF + PCI, PF and UPF and incident type 2 diabetes mellitus remained statistically significant after adjustment for WHtR or height, but the associations were attenuated by ∼50% with WHtR (Supplementary Table S8).

**Table 2:** The association between Nova group intake and incident type 2 diabetes mellitus.

Per 10%g/day increase	Quartile 1 (77,974)	Quartile 2 (77,972)	Quartile 3 (77,972)	Quartile 4 (77,974)	HR (95%CI)	p-value
**MPF + PCI**						
Cases/Non Cases	3384/74,590	3308/74,664	3450/74,522	4094/73,880	14,236/297,656	
Model 1	Reference	0.86 (0.82–0.90)	0.82 (0.78–0.86)	0.91 (0.87–0.96)	0.88 (0.87–0.89)	<0.001
Model 2	Reference	0.88 (0.84–0.93)	0.84 (0.79–0.88)	0.89 (0.84–0.94)	0.94 (0.92–0.96)	<0.001
Model 3	Reference	0.89 (0.85–0.94)	0.85 (0.80–0.90)	0.91 (0.86–0.96)	0.95 (0.93–0.97)	<0.001
Model 4	Reference	0.91 (0.86–0.96)	0.87 (0.82–0.92)	0.92 (0.87–0.97)	0.95 (0.94–0.97)	<0.001
Model 5	Reference	0.93 (0.88–0.97)	0.89 (0.84–0.94)	0.94 (0.89–1.00^a^)	0.96 (0.95–0.98)	<0.001
**PF**						
Cases/Non Cases	4314/73,660	3365/74,607	3162/74,810	3395/74,579	14,236/297,656	
Model 1	Reference	0.83 (0.79–0.86)	0.83 (0.79–0.86)	0.94 (0.89–0.98)	1.10 (1.08–1.11)	<0.001
Model 2	Reference	0.79 (0.76–0.83)	0.75 (0.71–0.79)	0.77 (0.72–0.83)	0.92 (0.89–0.95)	<0.001
Model 3	Reference	0.78 (0.75–0.82)	0.73 (0.69–0.77)	0.73 (0.68–0.79)	0.89 (0.86–0.92)	<0.001
Model 4	Reference	0.82 (0.78–0.86)	0.78 (0.74–0.83)	0.80 (0.75–0.86)	0.93 (0.90–0.95)	<0.001
Model 5	Reference	0.82 (0.78–0.86)	0.77 (0.73–0.82)	0.76 (0.71–0.82)	0.89 (0.87–0.92)	<0.001
**UPF**						
Cases/Non Cases	3883/74,091	3504/74,468	3302/74,670	3547/74,427	14,236/297,656	
Model 1	Reference	0.92 (0.88–0.97)	0.91 (0.86–0.95)	1.10 (1.05–1.15)	1.15 (1.13–1.18)	<0.001
Model 2	Reference	0.99 (0.95–1.04)	1.04 (0.98–1.09)	1.27 (1.20–1.33)	1.17 (1.14–1.19)	<0.001
Model 3	Reference	0.99 (0.94–1.04)	1.03 (0.98–1.09)	1.28 (1.21–1.35)	1.19 (1.16–1.21)	<0.001
Model 4	Reference	0.97 (0.92–1.02)	1.00 (0.95–1.05)	1.19 (1.13–1.26)	1.14 (1.11–1.16)	<0.001
Model 5	Reference	0.97 (0.92–1.02)	1.00 (0.95–1.05)	1.21 (1.14–1.28)	1.15 (1.13–1.18)	<0.001

Associations between Nova group intake and incident type 2 diabetes mellitus in EPIC. Nova group intakes expressed as a percentage of daily dietary intake (%g/day). Hazard ratios expressed per 10%g/day increase in Nova group intake, and across sex-specific quartiles. Cox proportional hazard models, with age as the underlying time variable. Time at entry was age at recruitment, and exit time was age at type 2 diabetes mellitus diagnosis, end of follow-up, loss to follow-up, or death, whichever came first. Model 1 was the unadjusted model. Model 2 was adjusted for sex, occupation, study centre, education level, smoking status and intensity, physical activity level, alcohol intake, family history of diabetes and total energy intake. Model 2 was further adjusted for saturated fat, sugar and sodium (model 3), Mediterranean Diet (model 4), saturated fat, sugar, sodium and Mediterranean Diet (model 5), and height (model 6).

95%CI: 95% confidence interval; HR: hazard ratio; MPF: unprocessed/minimally processed food; PCI: processed culinary ingredients; PF: processed food; SD: standard deviation; UPF: ultra-processed food.

a p-value = 0.049.

### Aim 2

Table 3 reports the substitution models replacing 10%g/day of dietary intake from one Nova group, with another. In adjusted models (model 2), replacing 10% g/day of UPF with MPF + PCI or PF was associated with lower incident type 2 diabetes mellitus (UPF replaced with: MPF + PCI HR per 10% g/day: 0.86 (95%CI: 0.84–0.88); with PF HR per 10% g/day: 0.82 (95%CI: 0.79–0.85)). Replacing MPF + PCI with PF was also associated with lower incident type 2 diabetes mellitus (MPF + PCI replaced with PF HR per 10% g/day: 0.93 (95%CI: 0.91–0.96)). Substitutions were similar after adjustment for saturated fat, sugar, sodium and Mediterranean diet adherence (model 5).

**Table 3:** The substitution effect of replacing 10% g/day of dietary intake from one Nova group, with another Nova group.

Per 10% replacement	Replace UPF with MPF + PCI		Replace UPF with PF		Replace MPF + PCI with UPF		Replace MPF + PCI with PF	
	HR (95%CI)	p-value	HR (95%CI)	p-value	HR (95%CI)	p-value	HR (95%CI)	p-value
Model 2	0.86 (0.84–0.88)	<0.001	0.82 (0.79–0.85)	<0.001	1.16 (1.14–1.19)	<0.001	0.93 (0.91–0.96)	<0.001
Model 5	0.87 (0.85–0.89)	<0.001	0.80 (0.77–0.83)	<0.001	1.15 (1.12–1.18)	<0.001	0.90 (0.87–0.93)	<0.001

Substitution effect of replacing 10% g/day of daily dietary intake from one Nova group, with another Nova group in EPIC. Cox proportional hazard models, with age as the underlying time variable. Time at entry was age at recruitment, and exit time was age at type 2 diabetes mellitus diagnosis, end of follow-up, loss to follow-up, or death, whichever came first. Model 2 was adjusted for sex, occupation, study centre, education level, smoking status and intensity, physical activity level, alcohol intake, family history of diabetes and total energy intake. Model 2 was further adjusted for saturated fat, sugar, sodium and Mediterranean Diet (model 5).

95%CI: 95% confidence interval; HR: hazard ratio; MPF: unprocessed/minimally processed food; PCI: processed culinary ingredients; PF: processed food; SD: standard deviation; UPF: ultra-processed food.

### Aim 3

**Fig. 2:**
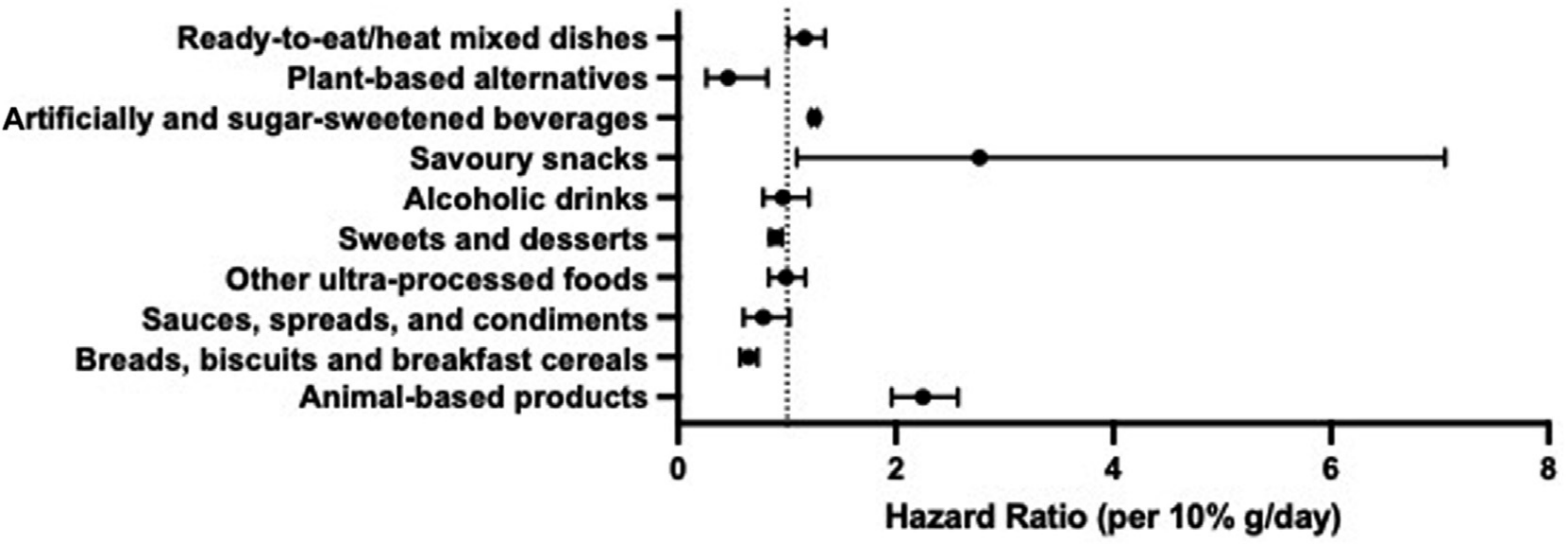
Hazard ratios and 95% confidence intervals of the association between UPF sub-group intake and incident type 2 diabetes mellitus. Associations between UPF sub-group intake and incident type 2 diabetes mellitus in EPIC. Sub-group intake expressed as a percentage of daily dietary intake (%g/day). Hazard ratios expressed per 10%g/day increase in sub-group intake. Cox proportional hazard models, with age as the underlying time variable. Time at entry was age at recruitment, and exit time was age at type 2 diabetes mellitus diagnosis, end of follow-up, loss to follow-up, or death, whichever came first. Model 2 was adjusted for sex, occupation, study centre, education level, smoking status and intensity, physical activity level, alcohol intake, family history of diabetes and total energy intake. Abbreviations: CI: confidence interval; HR: hazard ratio; SD: standard deviation; UPF: ultra-processed food.

Table 4 and Fig. 2 report associations between UPF sub-groups and incident type 2 diabetes mellitus. Each 10% g/day higher intake of savoury snacks (HR: 2.77 (95%CI: 1.09–7.05)), animal-based products (HR: 2.25 (95%CI: 1.96–2.57)), ready-to-eat/heat mixed dishes (HR: 1.16 (95%CI: 1.01–1.35)), and ASB/SSB (HR: 1.25 (95%CI: 1.22–1.28)) were associated with higher incident type 2 diabetes mellitus. Breads, biscuits and breakfast cereals (HR: 0.65 (95%CI: 0.57–0.73)), sweets and desserts (HR: 0.89 (95%CI: 0.84–0.95)), and plant-based alternatives (HR: 0.46 (95%CI: 0.26–0.82)) were associated with lower incident type 2 diabetes mellitus. Sauces, spreads, and condiments (p = 0.074); alcoholic drinks (p = 0.744) and other UPF (p = 0.870) were not associated with incident type 2 diabetes mellitus.

**Table 4:** The association between UPF sub-groups and incident type 2 diabetes mellitus.

Per 10%g/day increase in the diet	Model 2 - All UPF sub-groups entered simultaneously into model 2	
	HR (95%CI)	p-value
UPF (%g/day)		
Breads, biscuits and breakfast cereals	0.65 (0.57–0.73)	<0.001
Sauces, spreads, and condiments	0.78 (0.60–1.02)	0.074
Sweets and desserts	0.89 (0.84–0.95)	<0.001
Savoury Snacks	2.77 (1.09–7.05)	0.032
Plant-based alternatives	0.46 (0.26–0.82)	0.009
Animal-based products	2.25 (1.96–2.57)	<0.001
Ready-to-eat/heat mixed dishes	1.16 (1.01–1.35)	0.043
Artificially and sugar-sweetened beverages	1.25 (1.22–1.28)	<0.001
Alcoholic drinks	0.96 (0.78–1.20)	0.744
Other ultra-processed foods	0.99 (0.83–1.17)	0.870

Associations between UPF sub-group intake and incident type 2 diabetes mellitus in EPIC. Sub-group intake expressed as a percentage of daily dietary intake (%g/day). Hazard ratios expressed per 10%g/day increase in sub-group intake. Cox proportional hazard models, with age as the underlying time variable. Time at entry was age at recruitment, and exit time was age at type 2 diabetes mellitus diagnosis, end of follow-up, loss to follow-up, or death, whichever came first. Model 2 was adjusted for sex, occupation, study centre, education level, smoking status and intensity, physical activity level, alcohol intake, family history of diabetes and total energy intake.

95%CI: 95% confidence interval; HR: hazard ratio; SD: standard deviation; UPF: ultra-processed food.

### Aim 4

Table 5 reports the mediation analyses for WHtR. Under strong assumptions, WHtR mediated 46.4% (38.3–54.4) of the association between UPF and incident type 2 diabetes mellitus.

**Table 5:** Mediation analysis of the association between UPF intake and incident type 2 diabetes mellitus, for indicators of adiposity.

Adiposity indicator	Effect	Per 10%g/day increment in UPF	
		Estimate (95%CI	p-value
WHtR (n = 288,884)	Total Effect (TE)	1.14 (1.11–1.17)	<0.001
	Pure Natural Direct Effect (PNDE)	1.07 (1.05–1.10)	<0.001
	Total Natural Indirect Effect (TNIE)	1.06 (1.06–1.06)	<0.001
	Proportion Mediated (PM) %	46.4 (38.3–54.4)	<0.001

Model 2 was adjusted for sex, occupation, study centre, education level, smoking status and intensity, physical activity level, alcohol intake, family history of diabetes and total energy intake.

95%CI: 95% confidence interval; UPF: ultra-processed food; WHtR: waist-height-ratio.

### Sensitivity analyses

Results were largely unchanged in sensitivity analyses, including for upper- and lower-bound processing scenarios (Supplementary Fig. S7, Tables S9–S15, and described in Supplementary materials), except that UPF was not statistically significantly associated with incident type 2 diabetes mellitus in France or Italy (Supplementary Table S10). When modelled as kcal/day, %kcal/day or g/day, MPF + PCI was not associated with lower incident type 2 diabetes mellitus (Supplementary Table S13).

E-values for UPF and incident type 2 diabetes mellitus were 1.61 for the point estimate, and 1.54 for the 95%CI. This is the minimum strength of association (as a HR) that an unmeasured confounder would need to have with UPF and incident type 2 diabetes mellitus to fully explain the association, conditional on the measured covariates. For MPF + PCI, E-values were 1.33 for the point estimate and 1.26 for the 95%CI, and for PF, 1.40 for the point estimate and 1.30 for the 95%CI.

## Discussion

In this large-scale, prospective analysis across eight European countries, greater UPF intake was associated with higher incident type 2 diabetes mellitus. In contrast, greater intake of lower degrees of food processing was associated with lower incident type 2 diabetes mellitus. The associations between Nova groups and incident type 2 diabetes mellitus were not fully explained by nutrients (saturated fat, sodium (excluding discretionary sodium) and sugar) or diet quality. Replacing 10% g/day of UPF with MPF + PCI or PF was associated with lower incident type 2 diabetes mellitus. Replacing MPF + PCI with PF was also associated with lower incident type 2 diabetes mellitus. However, there was heterogeneity in the association between UPF subgroups and incident type 2 diabetes mellitus. Whilst some UPF sub-groups were associated with higher incident type 2 diabetes mellitus (ASB/SSBs, savoury snacks, animal-based products and ready-to-eat/heat dishes), others were inversely associated (breads, biscuits and breakfast cereals, sweets and desserts, and plant-based alternatives), or not associated (sauces, spreads, and condiments, alcoholic drinks, and other UPFs).

This is the first prospective study to examine the association between MPF + PCI, PF and UPF intake and incident type 2 diabetes mellitus. Regarding UPF, our results are in line with an umbrella review of meta-analyses indicating positive associations between UPF intake and 32 health outcomes, including type 2 diabetes mellitus.^16^ For type 2 diabetes mellitus, eight out of nine studies across eleven prospective cohorts have demonstrated increased risks associated with higher UPF intake.^14^^,^^37^^,^^38^ Whilst not statistically significant, the results from France and Italy in this study were directionally consistent and of similar magnitude to estimates from other EPIC countries. UPF intakes reported in some countries within EPIC are similar to, or lower than intakes reported in other studies, such as the Netherlands and France.^10^ This may be as baseline data collected from EPIC is from the 1990s, where diets may be more based on fresh foods.^22^ One previous study demonstrated a lower risk of type 2 diabetes mellitus with greater MPF intake (%g/day) in the US.^19^ Similarly in this current European analysis, higher MPF + PCI intakes were associated with lower incident type 2 diabetes mellitus, as was PF. No prospective studies to date have conducted substitution analyses between food processing groups and incident type 2 diabetes mellitus. For the first time, we demonstrate that replacing UPF with other lower degrees of food processing are associated with lower incident type 2 diabetes mellitus. The magnitude of the associations across the highest vs. lowest consumers of MPF + PCI, PF and UPF intake reported in this study are similar to the magnitudes reported in previous EPIC studies regarding diet and incident type 2 diabetes mellitus,^39^ including for Mediterranean diet adherence,^40^ and across other prospective cohort studies regarding Mediterranean diet adherence^41^^,^^42^ and food group intake^43^ on type 2 diabetes mellitus risk, that are considered clinically relevant.

There are several potential mechanisms linking greater UPF intake and incident type 2 diabetes mellitus. These can be considered as those regarding nutrient/diet quality largely captured in existing dietary guidance, and those resulting from ultra-processing^18^ (with some overlap). These mechanisms include nutrient and energy content/density, displacement of healthy foods and lower adherence to national public health dietary guidance, food matrix degradation, altered texture, taste and satiety,^6^^,^^44^^,^^45^ adverse effects of preservatives, neo-formed contaminants, additives and colours^18^^,^^46^ (such as inflammation^6^^,^^12^^,^^20^), dysregulated mechanisms of weight regulation and weight gain, and behavioural and environmental aspects such as hyper-palatability, marketing, low cost, large portion size, high availability and convenience.^18^^,^^46^^,^^47^

As in this study, previous studies found that adjusting for diet quality^15^^,^^18^^,^^19^^,^^48^, ^49^, ^50^ or fat intake,^51^ did not explain away the association between UPF and type 2 diabetes mellitus. One previous study found that nutrients explained only a small proportion (∼10%) of the association between UPF and type 2 diabetes mellitus.^15^ In this study, independent of a Mediterranean diet, higher UPF intake was still associated with higher incident type 2 diabetes mellitus. This was similarly seen in the US, where individuals had increased risks of type 2 diabetes mellitus with higher UPF intake, independent of the Alternative Healthy Eating Index.^15^

UPF intake may be linked with type 2 diabetes mellitus through greater adiposity.^52^ In this analysis, the association between UPF and incident type 2 diabetes mellitus was attenuated after adjustment for WHtR. Similar findings have been reported across previous cohorts, with attenuated estimates after adjustment for BMI.^15^^,^^19^^,^^48^^,^^49^^,^^51^^,^^53^ Under strong assumptions, WHtR potentially mediated 46.4% of the association between UPF and incident type 2 diabetes mellitus.

Given prior evidence linking specific UPFs such as processed meat or SSBs with type 2 diabetes mellitus^54^^,^^55^ and the improved nutritional profile of some UPFs,^20^ it is plausible that the association between UPF and type 2 diabetes mellitus is driven by specific UPF. Indeed, whilst some UPFs were associated with higher incident type 2 diabetes mellitus in this analysis, others were associated with a neutral, or reduced risk. Ultra-processed savoury snacks, animal-based products, ready-to-eat meals, and ASB/SSB were each associated with higher risk of incident type 2 diabetes mellitus. In contrast, ultra-processed breads, biscuits and breakfast cereals, sweets and desserts and plant-based alternatives were each associated with lower incident type 2 diabetes mellitus. Chen et al., also found that ASB and SSB, animal-based products and ready meals were associated with increased type 2 diabetes mellitus risk, whilst breads, cereals and sweets and desserts were associated with lower risk.^15^ However, they found that savoury snacks were associated with a decreased, not increased risk, and sauces, spreads and condiments and other UPFs were associated with an increased risk of type 2 diabetes mellitus. Similarly, Duan et al., identified UPF dietary patterns associated with opposing type 2 diabetes mellitus risks in the Netherlands.^49^ Warm savoury snack patterns (fried snacks, fries, and snack sauce) and cold savoury snack patterns (cheese, deli meat, and savoury spreads for crackers or French bread) were associated with an increased risk, whereas a sweet snack pattern (sweet biscuits/cookies, pastries, and chocolate) was associated with a decreased risk, and a traditional Dutch cuisine pattern (sliced bread, lunch meat, and gravy) was not associated with type 2 diabetes mellitus risk.^49^

Potential mechanisms linking UPF and type 2 diabetes mellitus, such as nutritional characteristics, energy density, hyperpalatability, additive content and the extent of matrix degradation can vary greatly across UPF sub-groups.^20^ The heterogeneity of these properties across UPF sub-groups may potentially explain the varying associations of sub-groups with incident type 2 diabetes mellitus. Increased consumption of soft drinks, typically lacking in micronutrients and fibre, may have a detrimental effect on incident type 2 diabetes mellitus, compared with fortified breads or breakfast cereals, which may contain some fibre. Some UPF were inversely associated with incident type 2 diabetes mellitus, including sweets and desserts. Duan et al. reported that the inverse association between a UPF sweet snack pattern and risk of type 2 diabetes mellitus could be a result of reverse causality.^49^ Furthermore, a previous EPIC study reported inverse associations between cakes and cookies, and incident type 2 diabetes mellitus,^56^ where it could not be ruled out that the observation was artefact.

In this study, a higher PF intake was associated with lower incident type 2 diabetes mellitus, where around 30–50% of PF consumed by participants was beer and wine. A previous EPIC study reported that moderate alcohol consumption, particularly wine and fortified wine, was associated with lower incident type 2 diabetes mellitus, in an inverted-U shaped association.^57^ This inverse association was stronger in females,^57^ who make up nearly two thirds of the sample in this study. This may explain the inverse association between PF and incident type 2 diabetes mellitus reported here, and the inverted-U profile of the restricted cubic spline plot for PF. PF also includes yoghurts, breads, and preserved fruits and vegetables, which have been previously shown to be associated with a lower risk of type 2 diabetes mellitus.^42^ But, PF also includes salted, smoked or canned meat, which would be expected to have a detrimental association.^42^ However, these meat products only constituted around 3% of PF intake in this study.

The potential for unmeasured confounding cannot be ruled out. Based on the E-value for the association between UPF intake and incident type 2 diabetes mellitus, an unmeasured confounder would need at least a moderate strength of association with the exposure and outcome, conditional on measured covariates, to explain away the exposure-outcome association. However, an unmeasured confounder with a smaller strength of association could explain the observation for MPF + PCI and PF with incident type 2 diabetes mellitus, suggesting greater confidence in the magnitude of the association for UPF.

Strengths of this study include the large multi-country sample, individual-level data, and long follow-up. The ability to differentiate between type 1 and type 2 diabetes mellitus is another key strength, with type 2 diabetes mellitus cases verified using multiple sources. An international team of experts performed the Nova coding.^22^ In addition, beyond assessing UPF intake and incident type 2 diabetes mellitus, several analyses using causal inference methods and considering sub-groups were conducted, which many studies have not performed. This is also one of the first cohort studies to examine health outcomes associated with all degrees of processing. A range of sensitivity analyses were conducted, with similar results to the main analysis, giving confidence in the results. However, the association for MPF + PCI differed with alternative units of exposure.

Several limitations must also be acknowledged. First, despite comprehensive efforts to standardise clinical incidences of type 2 diabetes mellitus, different centres had different information available locally for identifying and verifying cases, potentially introducing some heterogeneity.^30^ Second, information on diet and covariates was collected at baseline only. Diet and other behaviours impacting type 2 diabetes mellitus risk such as smoking, alcohol intake and physical activity can vary over time, potentially introducing residual confounding. Third, dietary information was collected before development of the Nova classification. Therefore, assumptions were made when insufficient information about processing was available from dietary assessments. Food processing may have changed, and UPF availability increased, since baseline. To overcome this, a sub-sample completing 24-h recalls was used to help inform the assumptions made on processing, and for misclassification.^22^ Furthermore, whilst extensive efforts were made to code the standardised database of >11,000 items in the ENDB with a uniform approach, the dietary assessment methods used across centres varied, potentially introducing discrepancies in measurement of UPF intake across centres. Three scenarios were therefore developed regarding the extent of food processing to account for potential misclassification of dietary intake into the Nova classification.^22^ The repeated results with upper- and lower-bound scenario estimates did not change the results, indicating the influence of any bias form misclassification did not alter results. Fourth, most EPIC participants are of European descent, limiting generalisability to other populations. Fifth, substitution analyses assume a linear relationship between the exposure and outcome, however PF tended to suggest a non-linear relationship.

### Conclusion

This study revealed associations between different degrees of food processing and incident type 2 diabetes mellitus. Higher UPF intake was associated with higher incident type 2 diabetes mellitus, whereas higher intakes of foods with less processing were associated with lower incident type 2 diabetes mellitus. These associations were robust to adjustment of diet or nutrient quality, and replacing UPF with other Nova groups was associated with lower incident type 2 diabetes mellitus. However, some UPF sub-groups were inversely associated with incident type 2 diabetes mellitus.

The value of incorporating ultra-processing within dietary guidance is debated,^7^^,^^58^^,^^59^ particularly regarding whether the effects of UPF can be explained with existing dietary knowledge.^7^^,^^18^ Whilst unmeasured confounding or measurement error cannot be ruled out, these sub-group analyses indicate significant heterogeneity within UPF. This questions the use of an overall UPF metric for public guidance, and supports recommendations to focus efforts on reducing consumption of specific UPF. Further work in cohorts with repeated dietary information is needed to confirm findings.

## Contributors

SJD, RLB, MJG, IH designed the analytical protocol, SJD performed the analyses and wrote the first draft of the manuscript. RLB, MJG and IH provided supervision. All authors were given the opportunity to comment on the analyses and provide comments on the manuscript. All authors have read and agreed to the final manuscript version.

## Data sharing statement

This study used EPIC data provided by EPIC centres. Details on how to access EPIC data and biospecimens are available at: https://epic.iarc.fr/access/index.php.

## Declaration of interests

SJD receives royalties from Amazon for a self-published book that mentions ultra-processed food, and payments from Red Pen Reviews. RLB is an employee of Eli Lilly and Company and reports honoraria from Novo Nordisk, Eli Lilly, Medscape, ViiV Healthcare Ltd and International Medical P and advisory board and consultancy work for Novo Nordisk, Eli Lilly, Pfizer, Gila Therapeutics Ltd, Epitomee Medical Ltd and ViiV Healthcare Ltd. All other authors declare no conflicts of interest.
