# Supplementary material for: Food consumption by degree of food processing and risk of type 2 diabetes mellitus: a prospective cohort analysis of the European Prospective Investigation into Cancer and Nutrition (EPIC)
Source: Lancet Reg Health Eur. 2024 Sep 16;46:101043. doi: 10.1016/j.lanepe.2024.101043 (PMC11551512; doi:10.1016/j.lanepe.2024.101043)
Supplement: Supplementary Figures S1–S7 and Tables S1–S15 [file mmc1.pdf]

Supplementary Materials: Food consumption by degree of food processing and risk of type 2 diabetes mellitus: a prospective cohort analysis of the European Prospective Investigation into Cancer and Nutrition (EPIC)

Supplementary Details:

1. Nova classification
2. UPF subgroups
3. Sodium calculation
4. Calculation of UK Eatwell guide adherence metric
5. Sensitivity Analyses

Supplementary Figures:

1. Flow chart of included participants.
2. Directed acyclic graph for the association between Nova groups and incident type 2 diabetes mellitus.
3. Flexible parametric model plots to test for proportional hazards.
4. Nova group intake across sex-specific quartiles of UPF intake.
5. Nova group intake by country.
6. Restricted cubic splines to test the assumption for linearity.
7. The association between Nova group intake and incident type 2 diabetes mellitus with upper-, middle- and lower-bound scenario estimates.

Supplementary Tables:

1. Dichotomised adherence cut-offs for Eatwell Guide adherence.
2. Ultra-processed food subgroups.
3. Characteristics of included vs. excluded participants.
4. Intake of MPF+PCI, PF and UPF by country.
5. Characteristics of participants across sex-specific quartiles of MPF+PCI and PF intake.
6. Intake of MPF+PCI, PF and UPF by food sub-group.
7. The association between Nova group intake and incident type 2 diabetes mellitus following adjustment for anthropometric and adiposity variables.
8. The association between Nova group intake and incident type 2 diabetes mellitus following adjustment for anthropometric and adiposity variables.
9. Significance values for interaction terms between country, sex, BMI, Mediterranean diet, energy intake and Nova group variables.
10. The association between Nova group intake and incident type 2 diabetes mellitus by country.
11. The association between Nova group intake and incident type 2 diabetes mellitus by subgroups of covariates with significant interaction terms with Nova group variables.
12. Sensitivity adjustments for the association between Nova group intake and incident type 2 diabetes mellitus.
13. The association between Nova group intake and incident type 2 diabetes mellitus with upper- and lower-bound scenario estimates, and with alternative Nova group metrics.
14. Dietary sensitivity adjustments for the association between Nova group intake and incident type 2 diabetes mellitus.
15. The association between MPF and PCI intake and incident type 2 diabetes mellitus as separate variables.

Supplementary Details

Nova classification *(adapted from Monteiro et al., 2019(1)).*

Minimally processed foods (MPF)

Unprocessed foods altered by industrial processes such as removal of inedible or unwanted parts, drying, crushing, grinding, fractioning, roasting, boiling, pasteurisation, refrigeration, freezing, placing in containers, vacuum packaging, non-alcoholic fermentation, and other methods that do not add salt, sugar, oils or fats or other food substances to the original food. The purpose of these processed are to: "preserve natural foods, to make them suitable for storage, or to make them safe or edible or more pleasant to consume"(1). Examples include fresh, squeezed, chilled, frozen, or dried fruits and vegetables; grains; legumes; meat, poultry, fish; eggs; milk; fruit or vegetable juices (with no added sugar, sweeteners or flavours); flakes or flour made from corn, wheat, oats, or cassava; seeds (with no added salt or sugar); herbs and spices, plain yoghurt; tea, coffee, and drinking water.

Processed culinary ingredients (PCI)

Substances obtained directly from minimally processed foods or from nature by industrial processes such as pressing, centrifuging, refining, extracting or mining. The purpose of PCIs is to make: "durable products that are suitable for use in home and restaurant kitchens to prepare, season and cook Group 1 foods and to make with them varied and enjoyable hand-made dishes and meals, such as stews, soups and broths, salads, breads, preserves, drinks and desserts" (1). They are used in preparing, seasoning and cooking minimally processed foods. Examples include vegetable oils; butter and lard; sugar and molasses; honey extracted; starches extracted from corn and other plants, and salt.

Processed food (PF)

Products made by adding salt, oil, sugar or other processed culinary ingredients to minimally processed foods, using preservation methods such as canning and bottling, or for breads and cheeses, using non-alcoholic fermentation. The purpose of processes and ingredients are used to increase the durability of minimally processed foods and make them more enjoyable, by modifying or enhancing sensory qualities. Examples include canned or bottled vegetables and legumes in brine; salted or sugared nuts and seeds; salted, dried, cured, or smoked meats and fish; canned fish; fruits in syrup and freshly made unpackaged breads and cheeses.

Ultra-processed food (UPF)

The purpose of UPFs is to make: "branded, convenient (durable, ready to consume), attractive (hyper-palatable) and highly profitable (low-cost ingredients) food products designed to displace all other food groups"(1). Formulations of ingredients mostly of exclusive industrial use, that result from a series of industrial processes. Many processes require sophisticated equipment and technology. Processes enabling the manufacture of ultra-processed foods include fractioning whole foods into substances, chemical modifications of substances, assembly of unmodified and modified food substances using industrial techniques such as hydrogenation, hydrolysation, extrusion, moulding and pre-frying, frequent application of additives whose function is to make the final product palatable or hyper-palatable ('cosmetic additives'), and sophisticated packaging, usually with synthetic materials(1). Ingredients often include sugar, oils and fats, and salt, generally in combination; substances that are sources of energy and nutrients but of no or rare culinary use such as high fructose corn syrup, hydrogenated or interesterified oils, and protein isolates; cosmetic additives such as flavours, flavour enhancers, colours, emulsifiers, sweeteners, thickeners, and anti-foaming, bulking, carbonating, foaming, gelling, and glazing agents; and additives that prolong product duration, protect original properties or prevent proliferation of microorganisms. Processes and ingredients used to manufacture ultra-processed foods are designed to create highly profitable (low cost and long shelf-life), convenient, hyper-palatable snacked products liable to displace all other NOVA food groups, notably minimally processed foods. Examples include carbonated soft drinks; sweet or savoury packaged snacks; chocolate, confectionery; ice-cream; mass-produced packaged breads and buns; margarines and other spreads; biscuits, pastries, cakes, and cake mixes; breakfast 'cereals', 'cereal' and 'energy' bars; 'energy' drinks; milk drinks, 'fruit' yoghurts and 'fruit' drinks; 'cocoa' drinks; 'instant' sauces; infant formulas, follow-on milks, other baby products; and 'health' and 'slimming' products such as meal replacement shakes and powders. Many ready to heat products including pre-prepared pies and pasta and pizza dishes; poultry and fish 'nuggets' and 'sticks',

sausages, burgers, hot dogs, and other reconstituted meat products, and powdered and packaged 'instant' soups, noodles and desserts are ultra-processed foods.

Sodium calculation

Discretionary sodium intake was not assessed in most of the EPIC dietary questionnaires. As a result, sodium intake in this analysis does not estimate (and underestimates) true sodium intake among the EPIC participants. Sodium intake in EPIC reflects sodium derived from food, while ignoring contributions from discretionary salt.

Calculation of the United Kingdom (UK) Eatwell guide adherence metric

The Eatwell Guide is a public health tool based on UK dietary guidelines. Adherence to the Eatwell Guide was constructed using a previously published metric using dichotomised adherence (yes or no, for 9 recommendations) for fruit and vegetables, fat, saturated fat, sugar, salt, fibre, red and processed meat, oily fish, and other fish (2).

Supplementary Table S1. Dichotomised adherence cutoffs for Eatwell Guide adherence, from UK dietary recommendations (3,4), and Scheelbeek et al., 2020 (2).

Eatwell Guide Component	Guidance
Total fat	35% of provided energy intake or below: ≤35% energy
Saturated fat	11% of provided energy intake or below, ≤11% energy
Salt	≤2363 mg/day sodium (i.e. 6g/day salt)
Carbohydrate	Around 50% of provided energy intake: ≥50% of energy
Total sugars	Less than 90g/2000kcal, <18% of total energy
Free sugars	<5% of total energy
Protein	Around 15% of provided energy intake: ≥14.5 & ≤15.5% of energy
Fibre	≥30g/day
Fruit and vegetables	Five portions per day*<br><br>*30g of dried fruit, max 150ml fruit juice or smoothie, and max 80g beans considered as one portion
Fish	≥20g per day
Oily Fish	≥20g per day
Red and processed meat	≤70g/day

*Fish recommendation: Fish ≥ 2 portions (2*140g) a week, one of which should be oily.*

Sensitivity Analyses

Models 2 and 5 were repeated with different covariates to assess the stability and confidence in results, including: without adjusting for occupation and/or for family history of type 2 diabetes mellitus (to check for any influence of missingness), additional adjustment for history of previous illness given the links between UPF and multi-morbidity[18], and for menopausal status and pill or hormone-replacement therapy use in females, given their influence on type 2 diabetes mellitus[37].

To further examine explanatory dietary factors, model 2 was adjusted for other nutrient variables linked with type 2 diabetes mellitus (fibre, and fruit and vegetables), all nutrients (protein, carbohydrate, sugar, total fat, saturated, monounsaturated and polyunsaturated fat, sodium), diet quality indices (Eatwell Guide, NutriScore and inflammatory score), and without adjusting for total energy. Models for PF and UPF were repeated, excluding alcohol from PF and UPF variables.

Alternate exposure variables include using upper- and lower-bound scenario estimates of Nova group intake (% g/day), intake expressed as percentage of daily energy intake (%kcal/day), and absolute intakes of weight and energy (grams/day and kcal/day). MPF and PCI were also examined separately to assess consistency in their association with incident type 2 diabetes mellitus.

Interactions

Interaction models indicated that there was no interaction effect between UPF intake and 1) sex and 2) Mediterranean diet, but there was an interaction effect with 1) BMI (p=0.007), 2) energy intake (p = 0.036) and 2) country (p<0.001) (Supplementary Table 6). Across countries, a greater intake of UPF was significantly associated with higher incident type 2 diabetes mellitus in Spain, the UK, The Netherlands, Germany, Sweden and Denmark, but not France or Italy (model 2) (Supplementary Table 7). MPF+PCI were significantly inversely associated with incident type 2 diabetes mellitus in the UK, Denmark and Germany. PF was significantly inversely associated with incident type 2 diabetes mellitus in The Netherlands, Germany, Sweden, and Denmark. Associations were largely unchanged with adjustment for saturated fat, sugar, sodium and Mediterranean diet adherence (model 5), except Italy, where UPF and MPF+PCI became significantly associated with higher incident type 2 diabetes mellitus; and in Italy and Spain, where PF became associated with lower incident type 2 diabetes mellitus. Little heterogeneity was observed across subgroups of BMI and energy intake (Supplementary Table 8).

Sensitivity adjustments

Results were largely unchanged across a number of sensitivity analyses (Supplementary Table 9); PF and UPF intake excluding alcohol, with complete case analysis, excluding the first 2 years of follow-up, excluding participants with cardiovascular disease or hypertension at baseline, without adjustment for occupation and/or without adjustment for family history of type 2 diabetes mellitus, with adjustment for history of previous illness, or with adjustment for menopausal status and pill or HRT use in females. Results were no longer significant for MPF+PCI intake after excluding participants with baseline cardiovascular disease and hypertension and with further adjustment for saturated fat, sugar, sodium content and Mediterranean diet adherence (model 5).

Repeating the main analysis using upper- and lower-bound scenarios for the level of processing did not significantly alter the associations between Nova groups and incident type 2 diabetes mellitus (Supplementary Table 10), with smaller effect sizes in the upper-bound scenario, and larger effect sizes in the lower-bound scenario. Results were similar for UPF when expressed in %kcal/day, g/day and kcal/day. MPF+PCI was associated with higher incident type 2 diabetes mellitus when expressed in g/day, but was not associated when expressed in %kcal/day and kcal/day. MPF+PCI was associated with higher incident T2MD when expressed in g/day, %kcal/day and kcal/day after adjustment for saturated fat, sugar, sodium content and Mediterranean diet adherence (model 5). Results were similar for PF when expressed in %kcal/day and kcal/day, but PF was not associated with incident type 2 diabetes mellitus when expressed in g/day. The association between MPF intake and PCI intake as separate variables and incident type 2 diabetes mellitus was directionally consistent with the main analyses for MPF+PCI.

Sensitivity dietary adjustments also largely unchanged results, including for EWG adherence, NutriScore, inflammatory score, fruit and vegetables, fibre, all nutrients combined, and without adjusting for total energy intake (Supplementary Table 11).

Supplementary Figures

Supplementary Figure S1. Flow chart of included participants.

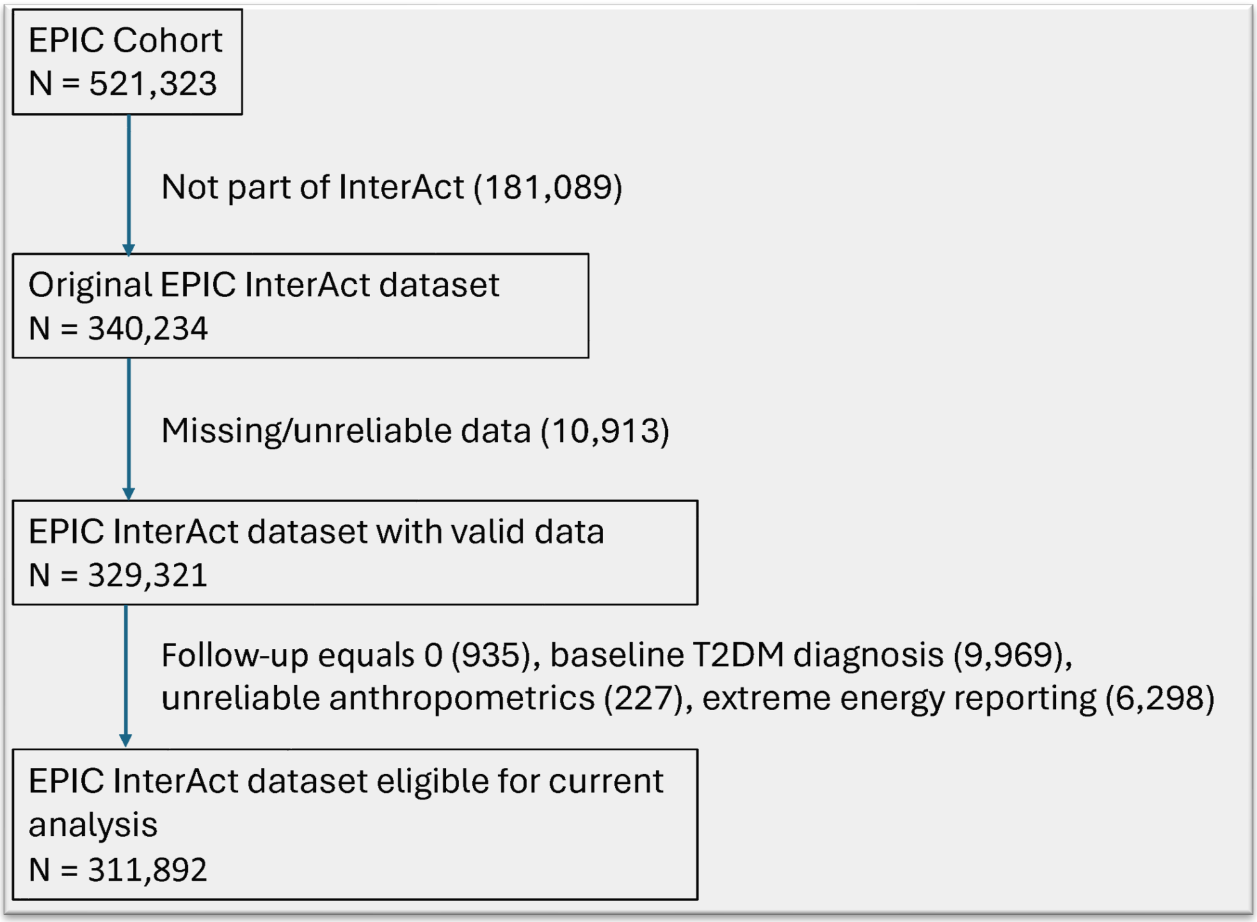

Abbreviations: EPIC: European Prospective Investigation into Cancer and Nutrition; T2DM: type 2 diabetes mellitus.

Supplementary Figure S2. Directed acyclic graph for the association between Nova groups and incident type 2 diabetes mellitus.

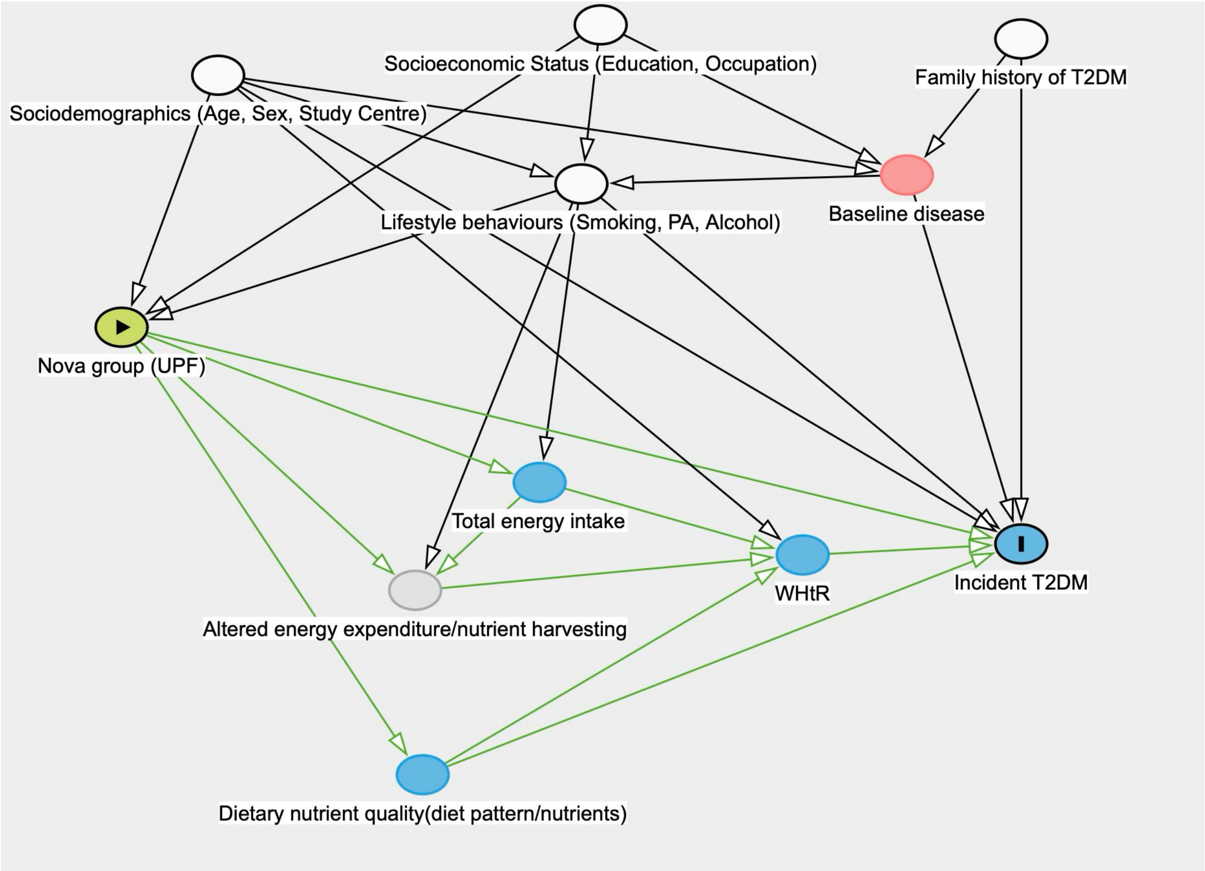

The adjustment set was identified by constructing a ***Directed acyclic graph (****DAG). Minimal sufficient adjustment sets containing Family history of type 2 diabetes mellitus, Lifestyle behaviours (Smoking, PA, Alcohol), Sociodemographics (Age, Sex, Study Centre), Socioeconomic Status (Education, Occupation) for estimating the total effect of Nova group (UPF) on Incident type 2 diabetes mellitus. Age was adjusted for as the underlying timescale. Total energy intake was adjusted for, to account for confounding influences of body size and dietary misreporting. Abbreviations: T2DM, type 2 diabetes mellitus, PA, physical activity, UPF, ultra-processed food; WHtR, waist-to-height ratio.*

Supplementary Figure S3. Flexible parametric model plots to test for proportional hazards.

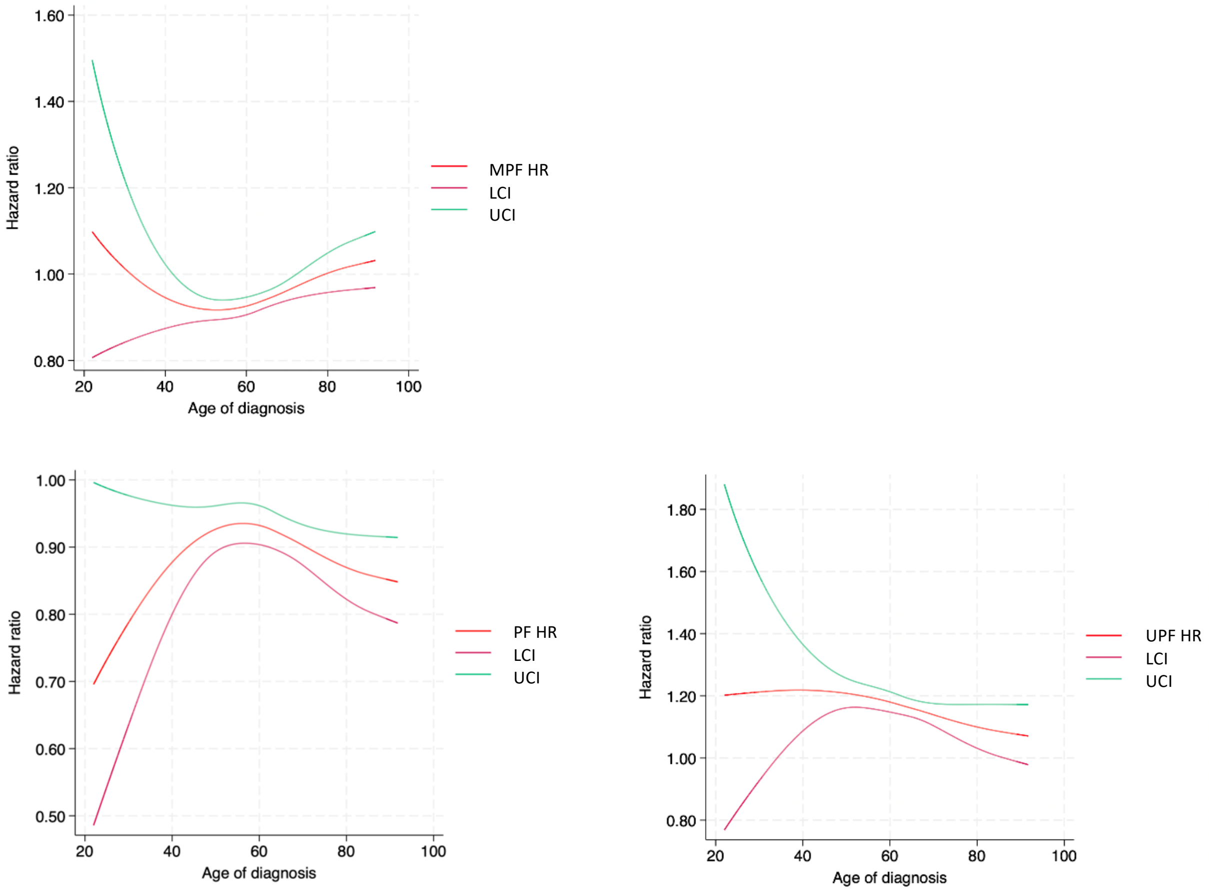

Flexible parametric models on the cumulative hazard scale, with restricted cubic splines with 5 internal knots to model the baseline hazard. Age was used as the time scale, with the NOVA variable modelled as a time-varying coefficient with 2 knots. HRs for a 10%g/day increase in intake were plotted against age at follow-up to determine the appropriateness of a summary HR over the follow-up duration. MPF and MPF+PCI variability is observed below age of 35 years (n=1,047) of beyond age 80 (n=8,539), where the sample is small (<10% of the total sample). The PH assumption is reasonably fulfilled across a large age range of follow-up and for the majority (>90%) of the sample. Abbreviations: LCI: lower confidence interval; HR: hazard ratio; MPF: unprocessed/minimally processed food; PCI: processed culinary ingredients; PF: processed food; UPF: ultra-processed food; UCI: upper confidence interval.

Supplementary Figure S4. Nova group intake across sex-specific quartiles of UPF intake.

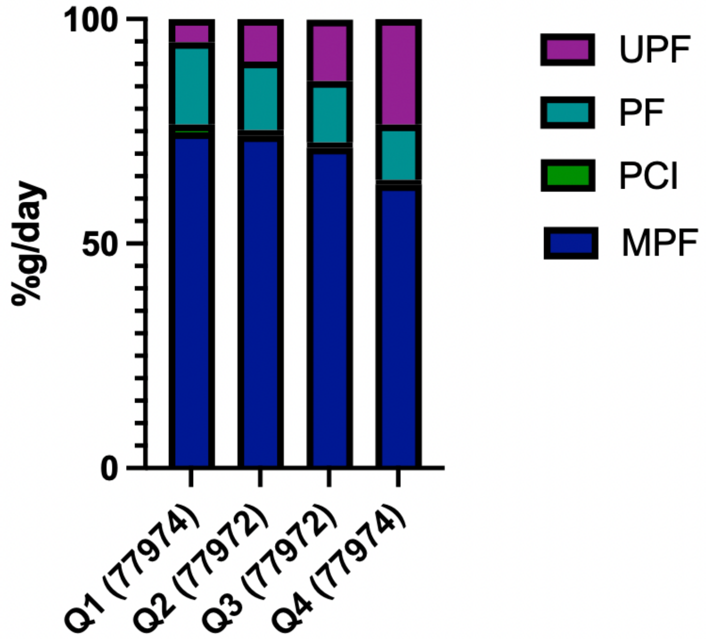

Abbreviations: MPF: unprocessed/minimally processed food; PCI: processed culinary ingredients; PF: processed food; Q: sex-specific quartile; UPF: ultra-processed food.

Supplementary Figure S5. Nova group intake by country.

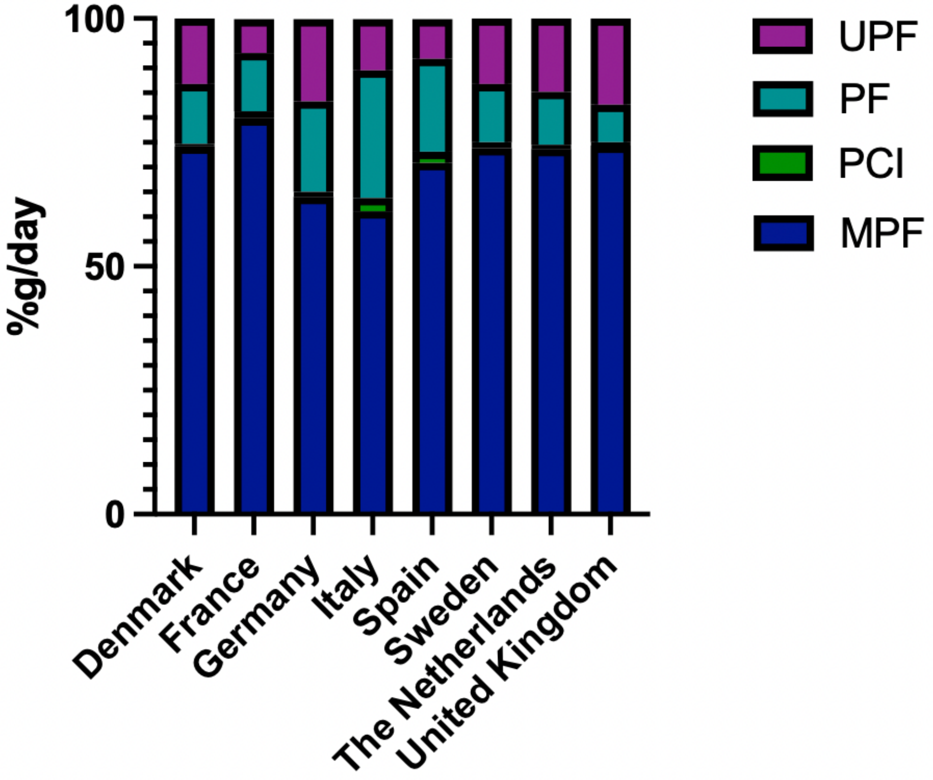

Abbreviations: MPF: unprocessed/minimally processed food; PCI: processed culinary ingredients; PF: processed food; Q: sex-specific quartile; UPF: ultra-processed food.

Supplementary Figure S6. Restricted cubic splines to test the assumption for linearity.

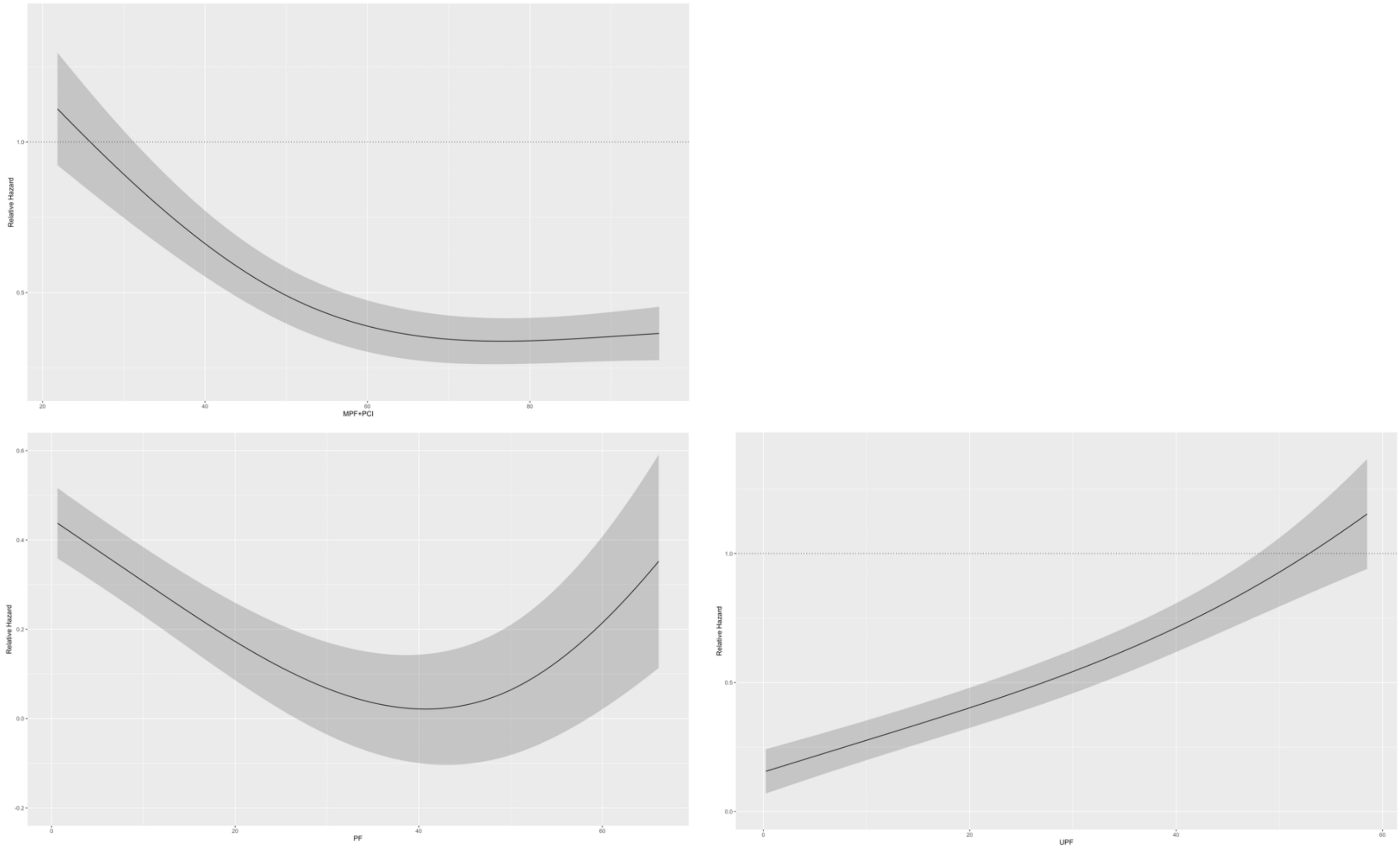

Top left: MPF+PCI; Bottom left: PF; Bottom-right: UPF. Non-linearity was assessed by modelling each Nova variable using restricted cubic splines. Adjusted for model 2 covariates, knots were placed at the 10th, 50th and 90th percentiles, and the relative hazard was then plotted against the respective Nova variable (%g /day). Abbreviations: MPF: unprocessed/minimally processed food; PCI: processed culinary ingredients; PF: processed food; UPF: ultra-processed food.

Supplementary Figure S7. The association between Nova group intake and incident type 2 diabetes mellitus with upper-, middle- and lower-bound scenario estimates.

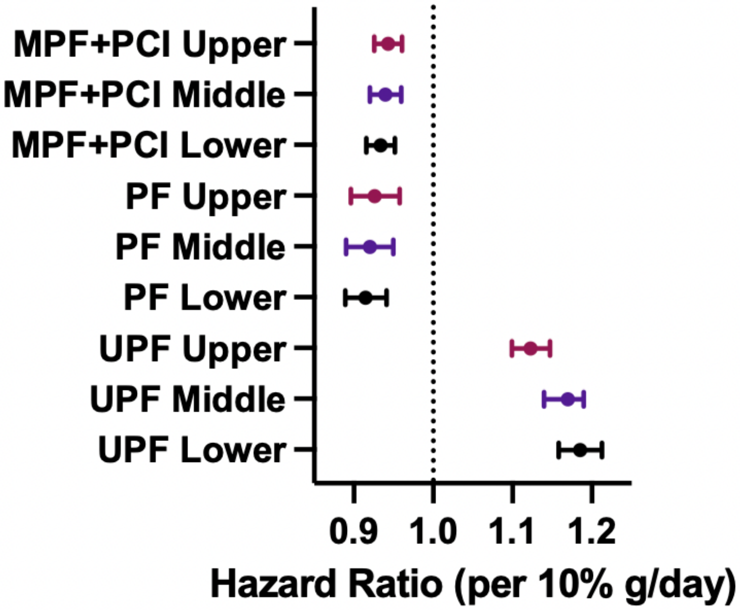

Nova group intakes expressed as a percentage of daily dietary intake (%g/day). Hazard ratios expressed per 10%g/day increase in Nova group intake. Cox proportional hazard models, with age as the underlying time variable. Time at entry was age at recruitment, and exit time was age at type 2 diabetes mellitus diagnosis, end of follow-up, loss to follow-up, or death, whichever came first. Model 2 was adjusted for sex, occupation, study centre, education level, smoking status and intensity, physical activity level, alcohol intake, family history of diabetes and total energy intake. Model 2 was further adjusted for saturated fat, sugar, sodium and Mediterranean Diet (model 5). Abbreviations: LB: lower bound; MB: middle bound; MPF: unprocessed/minimally processed food; PCI: processed culinary ingredients; PF: processed food; SD: standard deviation; UPF: ultra-processed food.

Supplementary Table S2. Ultra-processed food subgroups.

**UPF sub-group**	**Food and drink within sub-group**
Breads, biscuits and breakfast cereals	Breads<br>Biscuits<br>Breakfast cereals
Sauces, spreads, and condiments	Sauces, dressing and gravies - also in powder/dehydrated form/condensed form<br>Vegetable spread and products<br>Margarine
Sweets and desserts	Pastries, buns, and cakes<br>Ice cream, ice pops and frozen yogurts<br>Sweet snacks<br>Industrial desserts<br>Dairy desserts and drinks (ultra processed versions)
Savoury Snacks	Packaged salty snacks
Plant-Based Alternatives	Dairy substitute products<br>Meat alternatives
Animal-Based Products	Processed meat (beef, pork, and fish)<br>Processed cheese
Ready-to-eat/heat mixed dishes	Potato products<br>Pizza and focaccia (dough)<br>Pasta (filled)<br>Instant and canned soups<br>Ready meals<br>Vegetables and legumes in ultra-processed medium<br>Rice-based dishes
Artificially and sugar-sweetened beverages	Soft drinks<br>Fruit drinks, iced tea and other sweetened beverages
Alcoholic drinks	Alcoholic distilled drinks and other alcoholic drinks
Other ultra-processed foods	Artificial sweeteners<br>Alcohol-free versions of alcoholic beverages<br>Nutrition powders and drinks<br>Beverages dry weight

Supplementary Table S3. Characteristics of included vs. excluded participants.

**Variable**	**Total (329321)**	**Included (311892)**	**Excluded (17429)**	**p-value (Included vs. Excluded)**
UPF (%g/day)	13.0 (7.9)	13.0 (7.8)	12.6 (8.6)	<0.001
MPF+PCI (%g/day)	72.1 (12.2)	72.1 (12.1)	72.3 (12.9)	0.071
PF (%g/day)	14.9 (10.6)	14.9 (10.6)	15.1 (11.1)	0.090
Age (at baseline in years) (SD)	52.6 (9.4)	52.5 (9.4)	54.7 (9.2)	<0.001
Sex				<0.001
Men	120708 (37%)	113746 (36%)	6962 (40%)	
Women	208613 (63%)	198146 (64%)	10467 (60%)	
School				<0.001
None	14082 (4%)	12606 (4%)	1476 (8%)	
Primary school	99314 (30%)	92900 (30%)	6414 (37%)	
Technical/professional school	82442 (25%)	78809 (25%)	3633 (21%)	
Secondary school	54365 (17%)	51827 (17%)	2538 (15%)	
Longer education (incl. University degree)	71678 (22%)	68741 (22%)	2937 (17%)	
Not specified/Missing	7440 (2%)	7009 (2%)	431 (2%)	
Current occupation				<0.001
Employed	99374 (30%)	94764 (30%)	4610 (26%)	
Housewife	11096 (3%)	10553 (3%)	543 (3%)	
Retired	28089 (9%)	25546 (8%)	2543 (15%)	
Unemployed	7096 (2%)	6551 (2%)	545 (3%)	
Student	1084 (0.3%)	1023 (0.3%)	61 (0.3%)	
Other	2682 (1%)	2527 (1%)	155 (1%)	
Missing	179900 (55%)	170928 (55%)	8972 (51%)	
Smoking status				<0.001
Never	152299 (46%)	144105 (46%)	8194 (47%)	
Former	93514 (28%)	88481 (28%)	5033 (29%)	
Smoker	79845 (24%)	75877 (24%)	3968 (23%)	
Unknown	3663 (1%)	3429 (1%)	234 (1%)	
Physical activity level				<0.001
Inactive	55287 (17%)	53197 (17%)	2090 (12%)	
Moderately inactive	91726 (28%)	86808 (28%)	4918 (28%)	
Moderately active	123052 (37%)	116250 (37%)	6802 (39%)	

Active	29461 (9%)	28236 (9%)	1225 (7%)	
Missing	29795 (9%)	27401 (9%)	2394 (14%)	
Family History of type 2 diabetes mellitus (in parent or sibling)				<0.001
No	133212 (40%)	127751 (41%)	5461 (31%)	
Yes	30439 (9%)	28271 (9%)	2168 (12%)	
Missing	165670 (50%)	155870 (50%)	9800 (56%)	

Abbreviations: BMI: body mass index; MPF: unprocessed/minimally processed food; PCI: processed culinary ingredients; PF: processed food; SD: standard deviation; UPF: ultra-processed food; WC: waist circumference; WHtR: waist-to-height ratio.

Supplementary Table S4. Intake of MPF+PCI, PF and UPF by country.

%g/day	N	MPF+PCI (mean (SD))			PF (mean (SD))	UPF (mean (SD))
		MPF+PCI	MPF	PCI		
All	311892	72.1 (12.1)	70.8 (12.3)	1.3 (1.1)	14.9 (10.6)	13.0 (7.8)
Denmark	53568	74.6 (11.6)	74.2 (11.6)	0.5 (0.4)	12.1 (9.8)	13.2 (7.3)
France	19108	81.4 (7.5)	79.9 (7.6)	1.4 (0.6)	11.7 (6.1)	6.9 (4.2)
Germany	44360	65.1 (13.2)	64.0 (13.3)	1.0 (0.8)	18.3 (10.7)	16.6 (8.9)
Italy	42643	63.8 (11.0)	61.1 (10.8)	2.7 (1.0)	25.7 (10.4)	10.4 (6.5)
Spain	35571	73.1 (12.7)	70.9 (12.6)	2.2 (1.0)	18.8 (11.7)	8.0 (6.3)
Sweden	49415	75.0 (9.7)	73.9 (9.8)	1.1 (1.0)	11.8 (6.7)	13.2 (6.7)
The Netherlands	33379	74.5 (9.8)	73.7 (10.0)	0.8 (0.8)	10.7 (6.9)	14.8 (6.9)
United Kingdom	33848	75.0 (9.7)	74.4 (9.7)	0.6 (0.6)	7.6 (6.4)	17.4 (8.0)

Abbreviations: MPF: unprocessed/minimally processed food; PCI: processed culinary ingredients; PF: processed food; SD: standard deviation; UPF: ultra-processed food;

Supplementary Table S5. Characteristics of participants across sex-specific quartiles of MPF+PCI and PF intake.

		Characteristics of participants across sex-specific quartiles of MPF+PCI intake.					Characteristics of participants across sex-specific quartiles of PF intake.				
**Variable**	**All participants (311,892)**	**Quartile 1 (77974)**	**Quartile 2 (77972)**	**Quartile 3 (77972)**	**Quartile 4 (77974)**	**p-value**	**Quartile 1 (77974)**	**Quartile 2 (77972)**	**Quartile 3 (77972)**	**Quartile 4 (77974)**	**p-value**
%g											
MPF+PCI	72.1 (12.1)	56.7 (9.7)	70.0 (5.7)	77.1 (4.5)	84.6 (4.3)	<0.001	80.1 (9.0)	76.5 (8.7)	71.4 (9.3)	60.5 (11.3)	<0.001
MPF	70.8 (12.3)	55.2 (9.6)	68.6 (5.9)	76.0 (4.7)	83.6 (4.5)	<0.001	79.3 (9.1)	75.4 (8.7)	70.0 (9.2)	58.7 (11.2)	<0.001
PCI	1.3 (1.1)	1.5 (1.1)	1.4 (1.1)	1.2 (1.1)	1.0 (1.0)	<0.001	0.8 (0.9)	1.1 (1.0)	1.4 (1.1)	1.9 (1.2)	<0.001
PF	14.9 (10.6)	25.3 (13.1)	15.9 (7.7)	11.2 (5.3)	7.3 (3.6)	<0.001	5.2 (2.0)	9.9 (2.7)	15.8 (4.7)	28.8 (10.2)	<0.001
UPF	13.0 (7.8)	18.0 (10.7)	14.1 (6.5)	11.6 (4.9)	8.1 (3.8)	<0.001	14.7 (8.7)	13.6 (7.8)	12.8 (7.6)	10.7 (6.5)	<0.001
%kcal											
MPF+PCI	42.8 (12.5)	36.1 (10.8)	40.6 (11.0)	43.7 (10.8)	50.7 (12.5)	<0.001	45.2 (12.5)	43.0 (12.0)	41.8 (12.8)	41.2 (12.1)	<0.001
MPF	35.0 (10.4)	27.5 (7.7)	32.7 (8.1)	36.6 (8.5)	43.3 (10.1)	<0.001	40.0 (10.9)	36.4 (9.8)	33.4 (9.6)	30.4 (8.6)	<0.001
PCI	7.8 (6.3)	8.6 (6.0)	7.9 (6.3)	7.1 (6.2)	7.4 (6.7)	<0.001	5.2 (5.5)	6.6 (5.8)	8.5 (6.3)	10.8 (6.1)	<0.001
PF	26.2 (11.3)	33.6 (11.7)	27.6 (10.2)	23.9 (9.5)	19.6 (8.7)	<0.001	14.7 (6.3)	23.1 (7.1)	29.3 (7.8)	37.6 (8.9)	<0.001
UPF	31.0 (14.4)	30.3 (15.4)	31.8 (14.5)	32.4 (13.7)	29.7 (13.7)	<0.001	40.1 (13.4)	33.9 (12.4)	28.9 (12.7)	21.2 (11.8)	<0.001
Age (at baseline in years) (SD)	52.5 (9.4)	50.2 (9.4)	51.8 (9.6)	53.3 (9.2)	54.8 (8.6)	<0.001	54.4 (9.6)	52.6 (9.8)	51.8 (9.3)	51.2 (8.4)	<0.001
Sex											
Men	113746 (36%)	28437 (36%)	28436 (36%)	28436 (36%)	28437 (36%)		28437 (36%)	28436 (36%)	28436 (36%)	28437 (36%)	
Women	198146 (64%)	49537 (64%)	49536 (64%)	49536 (64%)	49537 (64%)		49537 (64%)	49536 (64%)	49536 (64%)	49537 (64%)	
School						<0.001					<0.001
None	12606 (4%)	3155 (4%)	3077 (4%)	2886 (4%)	3488 (4%)		1311 (2%)	2221 (3%)	3979 (5%)	5095 (7%)	
Primary school	92900 (30%)	25853 (33%)	22174 (28%)	21629 (28%)	23244 (30%)		23928 (31%)	20887 (27%)	20747 (27%)	27338 (35%)	
Technical/professional school	78809 (25%)	19147 (25%)	19875 (25%)	20318 (26%)	19469 (25%)		23819 (31%)	21374 (27%)	18853 (24%)	14763 (19%)	
Secondary school	51827 (17%)	12448 (16%)	13328 (17%)	13316 (17%)	12735 (16%)		11013 (14%)	13928 (18%)	13399 (17%)	13487 (17%)	
Longer education (incl. University degree)	68741 (22%)	16364 (21%)	17852 (23%)	17704 (23%)	16821 (22%)		13813 (18%)	18028 (23%)	20116 (26%)	16784 (22%)	
Not specified/Missing	7009 (2%)	1007 (1%)	1666 (2%)	2119 (3%)	2217 (3%)		4090 (5%)	1534 (2%)	878 (1%)	507 (1%)	
Current occupation						<0.001					<0.001
Employed	94764 (30%)	24275 (31%)	25790 (33%)	25026 (32%)	19673 (25%)		25444 (33%)	27671 (35%)	26245 (34%)	15404 (20%)	

Housewife	10553 (3%)	2376 (3%)	3153 (4%)	2923 (4%)	2101 (3%)		3926 (5%)	3033 (4%)	2327 (3%)	1267 (2%)	
Retired	25546 (8%)	4862 (6%)	6188 (8%)	6872 (9%)	7624 (10%)		9605 (12%)	6869 (9%)	5923 (8%)	3149 (4%)	
Unemployed	6551 (2%)	1970 (3%)	1672 (2%)	1529 (2%)	1380 (2%)		1718 (2%)	1830 (2%)	1838 (2%)	1165 (1%)	
Student	1023 (0.3%)	195 (0.3%)	282 (0.4%)	283 (0.4%)	263 (0.3%)		351 (0.5%)	374 (0.5%)	232 (0.3%)	66 (0.08%)	
Other	2527 (1%)	684 (1%)	727 (1%)	654 (1%)	462 (1%)		815 (1%)	823 (1%)	612 (1%)	277 (0.4%)	
Missing	170928 (55%)	43612 (56%)	40160 (52%)	40685 (52%)	46471 (60%)		36115 (46%)	37372 (48%)	40795 (52%)	56646 (73%)	
Height (cm)	166.8 (9.3)	166.2 (9.3)	166.9 (9.3)	167.2 (9.3)	166.7 (9.3)	<0.001	167.3 (9.1)	167.7 (9.3)	166.9 (9.3)	165.1 (9.2)	<0.001
BMI (kg/m2)	25.7 (4.1)	25.8 (4.2)	25.7 (4.1)	25.6 (4.0)	25.8 (4.1)	<0.001	25.9 (4.2)	25.5 (4.0)	25.6 (4.1)	25.9 (4.1)	<0.001
WC (cm)	85.4 (12.5)	85.5 (12.8)	85.2 (12.4)	85.2 (12.3)	85.6 (12.5)	0.901	85.6 (12.6)	85.0 (12.4)	85.1 (12.5)	85.8 (12.6)	<0.001
WHtR	0.5 (0.1)	0.5 (0.1)	0.5 (0.1)	0.5 (0.1)	0.5 (0.1)	<0.001	0.5 (0.1)	0.5 (0.1)	0.5 (0.1)	0.5 (0.1)	<0.001
Smoking status						<0.001					<0.001
Never	144105 (46%)	33825 (43%)	36089 (46%)	37162 (48%)	37029 (47%)		36210 (46%)	37510 (48%)	36997 (47%)	33388 (43%)	
Former	88481 (28%)	21378 (27%)	22387 (29%)	22637 (29%)	22079 (28%)		22293 (29%)	22542 (29%)	22241 (29%)	21405 (27%)	
Smoker	75877 (24%)	22279 (29%)	18722 (24%)	17135 (22%)	17741 (23%)		18070 (23%)	17080 (22%)	18021 (23%)	22706 (29%)	
Unknown	3429 (1%)	492 (1%)	774 (1%)	1038 (1%)	1125 (1%)		1401 (2%)	840 (1%)	713 (1%)	475 (1%)	
Smoking status and intensity						<0.001					<0.001
Never	130582 (42%)	30238 (39%)	32980 (42%)	33888 (43%)	33476 (43%)		34392 (44%)	34397 (44%)	33271 (43%)	28522 (37%)	
Current, 1-15 cigarettes/day	38770 (12%)	10740 (14%)	9849 (13%)	9060 (12%)	9121 (12%)		9390 (12%)	9300 (12%)	9515 (12%)	10565 (14%)	
Current, 16-25 cigarettes/day	21127 (7%)	6887 (9%)	5066 (6%)	4466 (6%)	4708 (6%)		5016 (6%)	4423 (6%)	4738 (6%)	6950 (9%)	
Current, 26+ cigarettes/day	5248 (2%)	2210 (3%)	1170 (2%)	900 (1%)	968 (1%)		999 (1%)	830 (1%)	1150 (1%)	2269 (3%)	
Former, quit <= 10 years	30818 (10%)	8256 (11%)	7850 (10%)	7453 (10%)	7259 (9%)		7048 (9%)	7522 (10%)	7749 (10%)	8499 (11%)	
Former, quit 11-20 years	27539 (9%)	7160 (9%)	7221 (9%)	6917 (9%)	6241 (8%)		6179 (8%)	6727 (9%)	7259 (9%)	7374 (9%)	
Former, quit 20+ years	26697 (9%)	5343 (7%)	6471 (8%)	7332 (9%)	7551 (10%)		7818 (10%)	7394 (9%)	6510 (8%)	4975 (6%)	
Current, pipe/cigar/occas	23089 (7%)	5769 (7%)	5361 (7%)	5650 (7%)	6309 (8%)		4124 (5%)	5269 (7%)	6035 (8%)	7661 (10%)	
Current/Former, missing	5086 (2%)	911 (1%)	1319 (2%)	1414 (2%)	1442 (2%)		1702 (2%)	1400 (2%)	1206 (2%)	778 (1%)	
Unknown	2936 (1%)	460 (1%)	685 (1%)	892 (1%)	899 (1%)		1306 (2%)	710 (1%)	539 (1%)	381 (0.5%)	
Physical activity level						<0.001					<0.001
Inactive	53197 (17%)	13897 (18%)	13417 (17%)	12937 (17%)	12946 (17%)		11551 (15%)	12531 (16%)	14615 (19%)	14500 (19%)	
Moderately inactive	86808 (28%)	21231 (27%)	21125 (27%)	21293 (27%)	23159 (30%)		21699 (28%)	20846 (27%)	22555 (29%)	21708 (28%)	

Moderately active	116250 (37%)	32404 (42%)	29263 (38%)	27281 (35%)	27302 (35%)		27874 (36%)	26506 (34%)	28261 (36%)	33609 (43%)	
Active	28236 (9%)	7507 (10%)	7283 (9%)	6898 (9%)	6548 (8%)		7721 (10%)	7195 (9%)	6501 (8%)	6819 (9%)	
Missing	27401 (9%)	2935 (4%)	6884 (9%)	8019 (10%)	27401 (9%)		9129 (12%)	10894 (14%)	6040 (8%)	1338 (2%)	
Family History of type 2 diabetes mellitus (in parent or sibling)						<0.001					<0.001
No	127751 (41%)	16680 (21%)	30302 (39%)	39108 (50%)	41661 (53%)		44963 (58%)	41035 (53%)	28868 (37%)	12885 (17%)	
Yes	28271 (9%)	3573 (5%)	6784 (9%)	8818 (11%)	9096 (12%)		10171 (13%)	9453 (12%)	6204 (8%)	2443 (3%)	
Missing	155870 (50%)	57721 (74%)	40886 (52%)	30046 (39%)	27217 (35%)		22840 (29%)	27484 (35%)	42900 (55%)	62646 (80%)	
Baseline cardiovascular disease, cancer, hypertension, hyperlipidaemia						<0.001					<0.001
No (for all)	103512 (33%)	32519 (42%)	24860 (32%)	22168 (28%)	23965 (31%)		19239 (25%)	19611 (25%)	26921 (35%)	37741 (48%)	
Yes (for at least one)	100430 (32%)	27847 (36%)	25459 (33%)	23499 (30%)	23625 (30%)		22735 (29%)	23432 (30%)	25843 (33%)	28420 (36%)	
Do Not Know (for at least one)	22407 (7%)	3832 (5%)	5036 (6%)	6190 (8%)	7349 (9%)		8184 (10%)	6785 (9%)	4619 (6%)	2819 (4%)	
Missing (none of above)	85543 (27%)	13776 (18%)	22617 (29%)	26115 (33%)	23035 (30%)		27816 (36%)	28144 (36%)	20589 (26%)	8994 (12%)	
Energy intake (kcal/day)	2121.8 (604.1)	2310.1 (632.3)	2194.0 (599.1)	2087.1 (570.8)	1895.9 (531.3)	<0.001	1971.0 (572.7)	2078.9 (582.8)	2159.2 (599.9)	2278.0 (617.8)	<0.001
Energy Density (kcal/100g)	83.1 (28.3)	100.7 (29.9)	88.1 (26.1)	77.9 (22.7)	65.8 (21.1)	<0.001	65.9 (19.1)	75.6 (21.0)	86.3 (25.3)	104.8 (30.3)	<0.001
Diet weight (g/day)	2758.9 (958.7)	2473.5 (936.6)	2650.1 (886.2)	2829.2 (888.5)	3082.7 (1010.4)	<0.001	3137.4 (951.4)	2887.8 (892.8)	2659.6 (889.4)	2350.7 (920.0)	<0.001
Alcohol (g/day)	13.3 (17.9)	23.3 (25.7)	14.0 (15.9)	9.9 (11.9)	6.0 (8.2)	<0.001	4.6 (6.7)	8.5 (9.8)	13.7 (14.4)	26.5 (25.8)	<0.001
Protein (g/day)	88.1 (26.9)	90.4 (27.7)	89.4 (27.1)	87.9 (26.3)	84.7 (25.9)	<0.001	84.6 (25.7)	86.4 (26.0)	88.5 (26.9)	93.1 (28.1)	<0.001
Carbohydrate (g/day)	234.4 (73.5)	251.5 (78.9)	241.6 (73.8)	232.1 (69.8)	212.5 (65.0)	<0.001	229.2 (73.5)	233.8 (71.7)	235.6 (72.5)	239.1 (75.7)	<0.001
Sugar (g/day)	105.4 (43.6)	108.4 (49.7)	107.7 (43.5)	105.5 (41.2)	100.0 (39.0)	<0.001	114.9 (48.1)	109.7 (43.9)	104.6 (41.6)	92.5 (37.1)	<0.001
Fibre (g/day)	22.7 (7.6)	21.6 (7.1)	23.0 (7.4)	23.4 (7.7)	23.1 (8.0)	<0.001	22.9 (8.1)	23.1 (7.5)	23.0 (7.4)	21.9 (7.2)	<0.001
Fat (g/day)	82.0 (28.6)	87.0 (29.2)	85.8 (28.8)	81.8 (28.0)	73.5 (26.4)	<0.001	75.9 (28.0)	82.1 (28.7)	85.3 (29.0)	84.9 (27.7)	<0.001
Saturated Fat (g/day)	32.0 (12.7)	33.3 (12.9)	33.4 (12.8)	32.3 (12.6)	28.9 (12.1)	<0.001	30.5 (12.8)	32.9 (12.9)	33.2 (12.9)	31.3 (12.0)	<0.001
Monounsaturated fat (g/day)	30.3 (12.3)	33.8 (12.8)	31.8 (12.6)	29.3 (11.7)	26.1 (10.8)	<0.001	26.0 (10.5)	28.7 (11.2)	31.5 (12.6)	34.9 (13.2)	<0.001
Polyunsaturated fat (g/day)	13.2 (5.8)	13.4 (6.0)	13.7 (5.9)	13.3 (5.7)	12.1 (5.4)	<0.001	12.9 (5.7)	13.5 (5.8)	13.6 (5.9)	12.6 (5.7)	<0.001
Sodium (mg/day)	2748.9 (1052.7)	2847.4 (1043.4)	2796.4 (1036.0)	2765.5 (1057.8)	2586.4 (1055.0)	<0.001	2688.9 (1095.6)	2742.1 (1064.9)	2764.7 (1025.9)	2800.0 (1019.5)	<0.001
Mediterranean Diet Adherence	8.0 (3.1)	8.2 (3.1)	8.1 (3.2)	7.9 (3.1)	7.9 (3.0)	<0.001	7.0 (2.9)	7.5 (2.9)	8.3 (2.9)	9.3 (3.1)	<0.001

NutriScore	5.9 (2.1)	6.5 (2.0)	6.2 (2.0)	5.9 (2.0)	5.2 (2.1)	<0.001	5.9 (2.3)	6.0 (2.0)	6.0 (2.0)	5.8 (1.9)	<0.001
Eatwell Guide Adherence (1-9)	3.2 (1.6)	3.0 (1.6)	3.1 (1.6)	3.2 (1.6)	3.6 (1.6)	<0.001	3.2 (1.6)	3.0 (1.6)	3.1 (1.6)	3.5 (1.6)	<0.001
Inflammatory Score of the Diet	0.6 (1.7)	1.0 (1.6)	0.6 (1.6)	0.5 (1.7)	0.4 (1.7)	<0.001	0.3 (1.8)	0.6 (1.7)	0.7 (1.6)	1.0 (1.5)	<0.001

Abbreviations: BMI: body mass index; MPF: unprocessed/minimally processed food; PCI: processed culinary ingredients; PF: processed food; SD: standard deviation; UPF: ultra-processed food; WC: waist circumference; WHtR: waist-to-height ratio.

Supplementary Table S6. Intake of MPF+PCI, PF and UPF by food sub-group.

	All participants (311892)	Quartile 1 (77974)	Quartile 2 (77972)	Quartile 3 (77972)	Quartile 4 (77974)
**%g/day in diet across sex-specific quartiles of MPF intake**	**Mean (SD)**	**Mean (SD)**	**Mean (SD)**	**Mean (SD)**	**Mean (SD)**
MPF (%g)	70.8 (12.3)	55.1 (9.6)	68.6 (5.8)	76.0 (4.6)	83.7 (4.4)
Water	10.2 (12.8)	4.8 (7.7)	8.8 (10.9)	11.4 (12.9)	15.6 (15.9)
Fruit	9.0 (8.0)	9.2 (7.8)	9.2 (8.2)	8.7 (7.7)	9.1 (8.3)
Milk and plain yoghurt	9.5 (8.0)	6.2 (6.2)	9.1 (7.5)	10.8 (8.2)	12.0 (8.9)
Cereal, grains and flour made from these foods	1.5 (1.9)	1.1 (1.3)	1.4 (1.7)	1.6 (2.0)	1.7 (2.3)
Potatoes	3.3 (2.7)	2.8 (2.2)	3.3 (2.6)	3.6 (2.9)	3.5 (3.0)
Pasta	1.8 (2.8)	2.8 (3.7)	2.0 (3.1)	1.3 (2.0)	0.9 (1.3)
Beans, lentils and chickpeas	0.8 (1.2)	0.8 (1.2)	0.8 (1.3)	0.8 (1.2)	0.7 (1.2)
Vegetables	6.4 (4.7)	6.0 (4.1)	6.5 (4.7)	6.4 (4.8)	6.5 (5.2)
Nuts and Seeds	0.1 (0.2)	0.1 (0.2)	0.1 (0.2)	0.1 (0.2)	0.04 (0.2)
Eggs	0.7 (0.7)	0.8 (0.7)	0.7 (0.7)	0.7 (0.7)	0.6 (0.6)
Poultry	0.8 (0.9)	0.9 (0.9)	0.8 (0.9)	0.8 (0.9)	0.7 (0.9)
Red meat	2.0 (1.5)	2.3 (1.7)	2.1 (1.6)	1.9 (1.4)	1.7 (1.3)
Fish	0.8 (1.1)	0.8 (1.0)	0.8 (1.1)	0.8 (1.1)	0.8 (1.2)
Seafood	0.2 (0.3)	0.2 (0.3)	0.1 (0.3)	0.1 (0.2)	0.1 (0.3)
Fungi	0.2 (0.2)	0.2 (0.2)	0.2 (0.2)	0.2 (0.3)	0.2 (0.3)
Coffee/tea	22.0 (13.7)	14.3 (10.5)	20.8 (12.6)	25.1 (13.2)	28.0 (14.4)
Fruit juice fresh and smoothies	0.4 (1.0)	0.4 (0.9)	0.4 (1.0)	0.4 (1.0)	0.4 (1.1)
Fruit juice fresh and smoothies	1.3 (2.3)	1.3 (2.4)	1.4 (2.4)	1.4 (2.3)	1.2 (2.2)
Homemade broth	0.1 (0.3)	0.1 (0.3)	0.1 (0.3)	0.1 (0.2)	0.1 (0.2)
**%g/day in diet across sex-specific quartiles of PCI intake**	**All participants (311892)**	**Quartile 1 (77974)**	**Quartile 2 (77972)**	**Quartile 3 (77972)**	**Quartile 4 (77974)**
PCI (%g)	1.3 (1.1)	0.2 (0.1)	0.6 (0.2)	1.4 (0.3)	2.9 (0.8)
Table sugar	0.5 (0.6)	0.1 (0.1)	0.2 (0.2)	0.5 (0.4)	1.1 (0.9)
Plant oil	0.5 (0.7)	0.05 (0.1)	0.2 (0.2)	0.5 (0.5)	1.4 (0.9)
Animal fats	0.3 (0.4)	0.1 (0.1)	0.2 (0.2)	0.4 (0.4)	0.4 (0.6)
Other processed culinary ingredients	0.02 (0.1)	0.006 (0.01)	0.02 (0.03)	0.04 (0.1)	0.03 (0.1)
Table salt	0.02 (0.03)	0.03 (0.03)	0.02 (0.03)	0.01 (0.02)	0.005 (0.01)
**%g/day in diet across sex-specific quartiles of PF intake**	**All participants (311892)**	**Quartile 1 (77974)**	**Quartile 2 (77972)**	**Quartile 3 (77972)**	**Quartile 4 (77974)**

PF (%g)	14.9 (10.6)	5.2 (2.0)	9.9 (2.7)	15.8 (4.7)	28.8 (10.2)
Cheese	1.5 (1.5)	0.8 (0.6)	1.2 (0.9)	1.6 (1.4)	2.3 (2.0)
Salted, smoked or canned meat, without additives	0.4 (0.8)	0.1 (0.3)	0.2 (0.5)	0.4 (0.8)	0.9 (1.2)
Salted, smoked or canned fish	0.3 (0.4)	0.2 (0.3)	0.3 (0.4)	0.3 (0.5)	0.4 (0.5)
Processed bread	4.0 (4.2)	1.1 (1.6)	3.0 (2.6)	4.7 (3.5)	7.3 (5.2)
Vegetables and other plant foods preserved	0.8 (1.4)	0.4 (0.5)	0.6 (0.7)	0.8 (1.1)	1.5 (2.3)
Legumes preserved	0.2 (0.7)	0.05 (0.2)	0.1 (0.3)	0.2 (0.5)	0.5 (1.2)
Fruit preserved	0.5 (0.8)	0.4 (0.5)	0.6 (0.7)	0.6 (0.9)	0.5 (0.9)
Nuts salted and nut spreads	0.1 (0.2)	0.1 (0.2)	0.1 (0.2)	0.1 (0.2)	0.1 (0.2)
Beer and Wine	6.4 (8.7)	1.3 (1.6)	3.3 (3.3)	6.4 (5.8)	14.5 (12.3)
Condensed milk, yogurt plain sweetened	0.2 (0.6)	0.2 (0.5)	0.3 (0.6)	0.2 (0.6)	0.1 (0.5)
Bread crumbs	0.02 (0.03)	0.01 (0.03)	0.01 (0.03)	0.01 (0.03)	0.02 (0.04)
Meringue, non ultra-processed bakeries	0.3 (0.7)	0.3 (0.7)	0.3 (0.8)	0.2 (0.5)	0.3 (0.6)
Sauce homemade, sweet or savoury	0.2 (0.4)	0.1 (0.2)	0.1 (0.2)	0.2 (0.4)	0.4 (0.6)
**%g/day in diet across sex-specific quartiles of UPF intake**	**All participants (311892)**	**Quartile 1 (77974)**	**Quartile 2 (77972)**	**Quartile 3 (77972)**	**Quartile 4 (77974)**
UPF (%g)	13.0 (7.8)	5.1 (1.9)	9.5 (1.5)	13.7 (1.8)	23.5 (7.2)
Breads, biscuits and breakfast cereals	1.9 (2.1)	1.0 (1.2)	1.9 (1.8)	2.2 (2.2)	2.4 (2.5)
Sauces, spreads, and condiments	0.9 (0.8)	0.4 (0.5)	0.9 (0.7)	1.1 (0.8)	1.3 (1.0)
Sweets and desserts	3.3 (3.1)	1.4 (1.2)	2.6 (1.8)	3.8 (2.5)	5.5 (4.4)
Savoury Snacks	0.1 (0.2)	0.02 (0.1)	0.05 (0.1)	0.1 (0.2)	0.1 (0.3)
Plant-Based Alternatives	0.1 (0.9)	0.006 (0.1)	0.02 (0.1)	0.03 (0.3)	0.2 (1.7)
Animal-Based Products	1.5 (1.3)	1.0 (0.9)	1.4 (1.1)	1.7 (1.3)	2.0 (1.6)
Ready-to-eat/heat mixed dishes	1.3 (1.4)	0.5 (0.7)	1.0 (1.0)	1.5 (1.4)	2.0 (1.9)
Artificially and sugar-sweetened beverages	3.4 (5.6)	0.4 (0.8)	1.3 (1.5)	2.8 (2.7)	9.3 (8.1)
Alcoholic drinks	0.3 (0.8)	0.2 (0.5)	0.3 (0.6)	0.4 (0.8)	0.4 (1.1)
Other ultra-processed foods	0.1 (1.0)	0.04 (0.2)	0.1 (0.3)	0.1 (0.6)	0.3 (1.8)

Abbreviations: MPF: unprocessed/minimally processed food; PCI: processed culinary ingredients; PF: processed food; SD: standard deviation; UPF: ultra-processed food;

Supplementary Table S7. The association between Nova group intake and incident type 2 diabetes mellitus following adjustment for anthropometric and adiposity variables.

%g/day in diet across sex-specific quartiles of Relative Fat Mass	All participants (311892)	Quartile 1 (72382)	Quartile 2 (72101)	Quartile 3 (72182)	Quartile 4 (72219)	Missing WC data (230008)
UPF (%g)	13.0 (7.8)	13.7 (7.9)	12.9 (7.7)	12.6 (7.7)	12.3 (8.3)	14.1 (6.9)
Breads, biscuits and breakfast cereals	1.9 (2.1)	2.1 (2.2)	2.1 (2.1)	1.9 (2.0)	1.7 (2.0)	1.1 (0.8)
Sauces, spreads, and condiments	0.9 (0.8)	1.0 (0.9)	0.9 (0.8)	0.8 (0.8)	0.7 (0.7)	1.8 (0.9)
Sweets and desserts	3.3 (3.1)	3.6 (3.3)	3.4 (3.2)	3.3 (3.2)	3.1 (3.2)	2.5 (1.8)
Savoury Snacks	0.1 (0.2)	0.1 (0.2)	0.1 (0.2)	0.1 (0.2)	0.1 (0.2)	0.1 (0.1)
Plant-Based Alternatives	0.1 (0.9)	0.2 (1.4)	0.1 (0.9)	0.04 (0.6)	0.02 (0.5)	0.0 (0.0)
Animal-Based Products	1.5 (1.3)	1.3 (1.2)	1.4 (1.2)	1.5 (1.2)	1.6 (1.3)	2.5 (1.4)
Ready-to-eat/heat mixed dishes	1.3 (1.4)	1.5 (1.5)	1.3 (1.5)	1.2 (1.4)	1.0 (1.4)	1.3 (1.0)
Artificially and sugar-sweetened beverages	3.4 (5.6)	3.4 (5.4)	3.2 (5.3)	3.3 (5.4)	3.5 (6.1)	4.7 (5.8)
Alcoholic drinks	0.3 (0.8)	0.3 (0.7)	0.3 (0.8)	0.3 (0.9)	0.3 (0.8)	0.1 (0.2)
Other ultra-processed foods	0.1 (1.0)	0.2 (1.0)	0.1 (1.0)	0.2 (1.1)	0.2 (1.1)	0.0 (0.1)

Abbreviations: UPF, ultra-processed food, WC, waist circumference

Supplementary Table S8. The association between Nova group intake and incident type 2 diabetes mellitus following adjustment for anthropometric and adiposity variables.

per 10%g/day increment	Cases/ Numbers	MPF+PCI				PF				UPF			
		Model 2 HR (95%CI)	Model 2 p-value	Model 5 HR (95%CI)	Model 5 p-value	Model 2 HR (95%CI)	Model 2 p-value	Model 5 HR (95%CI)	Model 5 p-value	Model 2 HR (95%CI)	Model 2 p-value	Model 5 HR (95%CI)	Model 5 p-value
Adjusting for WHtR	13487/288884	0.97 (0.95-0.99)	<0.001	0.98 (0.96-1.00)	0.023	0.96 (0.93-0.99)	0.006	0.95 (0.92-0.98)	<0.001	1.08 (1.06-1.11)	<0.001	1.08 (1.05-1.10)	<0.001
Adjusting for height	14236/311892	0.94 (0.92-0.96)	<0.001	0.97 (0.95-0.99)	0.002	0.92 (0.89-0.94)	<0.001	0.89 (0.86-0.91)	<0.001	1.16 (1.14-1.19)	<0.001	1.15 (1.13-1.18)	<0.001

Nova group intakes expressed as a percentage of daily dietary intake (%g/day). Hazard ratios expressed per 10%g/day increase in Nova group intake, and across sex-specific quartiles. Cox proportional hazard models, with age as the underlying time variable. Time at entry was age at recruitment, and exit time was age at type 2 diabetes mellitus diagnosis, end of follow-up, loss to follow-up, or death, whichever came first. Model 2 was adjusted for sex, occupation, study centre, education level, smoking status and intensity, physical activity level, alcohol intake, family history of diabetes and total energy intake. Model 2 was further adjusted for saturated fat, sugar, sodium and Mediterranean Diet (model 5). Abbreviations: 95%CI: 95% confidence interval; MPF: unprocessed/minimally processed food; PCI: processed culinary ingredients; PF: processed food; SD: standard deviation; UPF: ultra-processed food; WHtR: waist-to-height ratio.

Supplementary Table S9. Significance values for interaction terms between country, sex, body mass index, Mediterranean diet, energy intake and Nova group variables.

Covariate	Interaction with MPF+PCI intake (%g/day) (model 2)	Interaction with PF intake (%g/day) (model 2)	Interaction with UPF intake (%g/day) (model 2)
	**P-value**	**P-value**	**P-value**
Country	<0.001	<0.001	<0.001
Sex	0.041	<0.001	0.410
BMI	0.065	<0.001	0.007
Mediterranean diet	0.738	0.063	0.162
Energy intake	<0.001	0.001	0.036

Nova group intakes expressed as a percentage of daily dietary intake (%g/day). Interaction terms were added to model 2. Model 2 was adjusted for sex, occupation, study centre, education level, smoking status and intensity, physical activity level, alcohol intake, family history of diabetes and total energy intake. Abbreviations: BMI: body mass index; MPF: unprocessed/minimally processed food; PCI: processed culinary ingredients; PF: processed food; UPF: ultra-processed food.

Supplementary Table S10. The association between Nova group intake and incident type 2 diabetes mellitus by country.

Country	Cases/Numbers	MPF+PCI				PF				UPF			
per 10%g/day increment		Model 2 HR (95%CI)	Model 2 p-value	Model 5 HR (95%CI)	Model 5 p-value	Model 2 HR (95%CI)	Model 2 p-value	Model 5 HR (95%CI)	Model 5 p-value	Model 2 HR (95%CI)	Model 2 p-value	Model 5 HR (95%CI)	Model 5 p-value
Denmark	4491/53568	0.92 (0.89-0.95)	<0.001	0.98 (0.95-1.02)	<0.001	0.93 (0.87-0.98)	0.009	0.91 (0.86-0.96)	0.001	1.18 (1.13-1.23)	<0.001	1.08 (1.04-1.13)	<0.001
France	269/19108	1.00 (0.82-1.23)	0.989	1.10 (0.89-1.34)	0.383	0.82 (0.62-1.09)	0.167	0.76 (0.57-1.02)	0.066	1.23 (0.93-1.62)	0.140	1.10 (0.82-1.48)	0.504
Germany	1513/44360	0.95 (0.90-0.99)	0.031	0.94 (0.90-0.99)	0.025	0.90 (0.83-0.98)	0.014	0.91 (0.83-0.99)	0.029	1.16 (1.09-1.23)	<0.001	1.18 (1.11-1.27)	<0.001
Italy	1310/42643	1.00 (0.94-1.07)	0.978	1.09 (1.01-1.17)	0.031	0.95 (0.88-1.03)	0.192	0.75 (0.68-0.84)	<0.001	1.07 (0.98-1.17)	0.125	1.12 (1.02-1.24)	0.017
Spain	2456/35571	0.98 (0.93-1.02)	0.341	1.02 (0.97-1.08)	0.400	0.97 (0.92-1.03)	0.315	0.88 (0.82-0.94)	<0.001	1.09 (1.02-1.16)	0.010	1.11 (1.04-1.19)	0.002
Sweden	2456/49415	1.01 (0.96-1.06)	0.691	1.01 (0.97-1.07)	0.58	0.79 (0.73-0.86)	<0.001	0.78 (0.72-0.85)	<0.001	1.14 (1.07-1.21)	<0.001	1.14 (1.07-1.22)	<0.001
The Netherlands	797/33379	0.95 (0.85-1.05)	0.332	0.98 (0.88-1.10)	0.777	0.76 (0.64-0.90)	0.001	0.70 (0.58-0.84)	<0.001	1.23 (1.10-1.38)	<0.001	1.22 (1.09-1.38)	<0.001
United Kingdom	944/33848	0.76 (0.71-0.82)	<0.001	0.76 (0.71-0.82)	<0.001	0.84 (0.70-1.01)	0.069	0.84 (0.70-1.01)	0.065	1.40 (1.30-1.51)	<0.001	1.41 (1.31-1.52)	<0.001

Nova group intakes expressed as a percentage of daily dietary intake (%g/day). For each country, covariates that did not converge were included as stratifying variables. Hazard ratios expressed per 10%g/day increase in Nova group intake, and across sex-specific quartiles. Cox proportional hazard models, with age as the underlying time variable. Time at entry was age at recruitment, and exit time was age at type 2 diabetes mellitus diagnosis, end of follow-up, loss to follow-up, or death, whichever came first. Model 2 was adjusted for sex, occupation, study centre, education level, smoking status and intensity, physical activity level, alcohol intake, family history of diabetes and total energy intake. Model 2 was further adjusted for saturated fat, sugar, sodium and Mediterranean Diet (model 5). Abbreviations: 95%CI: 95% confidence interval; MPF: unprocessed/minimally processed food; PCI: processed culinary ingredients; PF: processed food; SD: standard deviation; UPF: ultra-processed food.

Supplementary Table S11. The association between Nova group intake and incident type 2 diabetes mellitus by subgroups of covariates with significant interaction terms with Nova group variables.

**Covariate Subgroup Analyses**	**Per 10% increment**	**MPF+PCI**				**PF**				**UPF**			
**per 10%g/day increment**	**Cases/Numbers**	**Model 2 HR (95%CI)**	**Model 2 p-value**	**Model 5 HR (95%CI)**	**Model 5 p-value**	**Model 2 HR (95%CI)**	**Model 2 p-value**	**Model 5 HR (95%CI)**	**Model 5 p-value**	**Model 2 HR (95%CI)**	**Model 2 p-value**	**Model 5 HR (95%CI)**	**Model 5 p-value**
BMI													
<25 Normal weight	2079/148193	0.93 (0.89-0.98)	0.009	0.95 (0.90-1.00)	0.043	0.99 (0.92-1.07)	0.775	0.97 (0.89-1.05)	0.413	1.13 (1.06-1.20)	<0.001	1.13 (1.05-1.21)	<0.001
25-30 Overweight	6289/121090	0.90 (0.87-0.92)	<0.001	0.91 (0.89-0.94)	<0.001	1.05 (1.00-1.09)	0.034	1.02 (0.98-1.06)	0.395	1.15 (1.11-1.19)	<0.001	1.15 (1.11-1.20)	<0.001
≥30 Obesity	5868/42609	0.92 (0.90-0.95)	<0.001	0.94 (0.91-0.96)	<0.001	1.02 (0.98-1.07)	0.260	1.01 (0.97-1.06)	0.542	1.11 (1.08-1.15)	<0.001	1.10 (1.06-1.14)	<0.001
Energy Intake (above median vs. median and below; median = 2109.8kcal/day)													
above median	7624/155934	0.94 (0.92-0.97)	<0.001	0.97 (0.94-1.00)	0.018	0.92 (0.89-0.95)	<0.001	0.89 (0.86-0.93)	<0.001	1.17 (1.13-1.21)	<0.001	1.16 (1.12-1.19)	<0.001
median and below	6612/155958	0.92 (0.89-0.94)	<0.001	0.95 (0.92-0.97)	<0.001	0.96 (0.92-1.01)	0.108	0.91 (0.87-0.96)	<0.001	1.17 (1.12-1.20)	<0.001	1.15 (1.11-1.19)	<0.001
Sex													
Males	7244/113746	0.96 (0.93-0.98)	<0.001	0.98 (0.95-1.00)	0.059	0.92 (0.89-0.95)	<0.001	0.90 (0.87-0.94)	<0.001	1.17 (1.13-1.20)	<0.001	1.15 (1.11-1.19)	<0.001
Females	6992/198146	0.92 (0.90-0.95)	<0.001	0.96 (0.93-0.98)	0.003	0.91 (0.86-0.96)	<0.001	0.83 (0.79-0.88)	<0.001	1.17 (1.13-1.21)	<0.001	1.16 (1.12-1.20)	<0.001

Nova group intakes expressed as a percentage of daily dietary intake (%g/day). Hazard ratios expressed per 10%g/day increase in Nova group intake. Cox proportional hazard models, with age as the underlying time variable. Time at entry was age at recruitment, and exit time was age at type 2 diabetes mellitus diagnosis, end of follow-up, loss to follow-up, or death, whichever came first. Model 2 was adjusted for sex, occupation, study centre, education level, smoking status and intensity, physical activity level, alcohol intake, family history of diabetes and total energy intake. Model 2 was further adjusted for saturated fat, sugar, sodium and Mediterranean Diet (model 5). Abbreviations: 95%CI: 95% confidence interval; MPF: unprocessed/minimally processed food; PCI: processed culinary ingredients; PF: processed food; SD: standard deviation; UPF: ultra-processed food.

Supplementary Table S12. Sensitivity adjustments for the association between Nova group intake and incident type 2 diabetes mellitus.

**Sensitivity Adjustment**	**Cases/Numbers**	**MPF+PCI**				**PF**				**UPF**		
**per 10%g/day increment**		**Model 2 HR (95%CI)**	**Model 2 p-value**	**Model 5 HR (95%CI)**	**Model 5 p-value**	**Model 2 HR (95%CI)**	**Model 2 p-value**	**Model 5 HR (95%CI)**	**Model 5 p-value**	**Model 2 HR (95%CI)**	**Model 2 p-value**	**Model 5 HR (95%CI)**
Complete Cases	4134/110574	0.95 (0.92-0.98)	0.002	0.95 (0.92-0.98)	0.004	0.86 (0.81-0.91)	<0.001	0.84 (0.80-0.89)	<0.001	1.19 (1.14-1.24)	<0.001	1.21 (1.16-1.26)
Exclude first 2 years of follow-up	12947/309002	0.94 (0.92-0.96)	<0.001	0.96 (0.94-0.98)	<0.001	0.92 (0.89-0.95)	<0.001	0.89 (0.86-0.92)	<0.001	1.17 (1.14-1.20)	<0.001	1.16 (1.13-1.19)
Adjusting for baseline illness: cardiovascular disease (stroke, angina, myocardial infarction), cancer, hypertension and hyperlipidaemia	14236/311892	0.94 (0.92-0.96)	<0.001	0.96 (0.94-0.98)	<0.001	0.93 (0.91-0.96)	<0.001	0.91 (0.88-0.94)	<0.001	1.16 (1.13-1.19)	<0.001	1.15 (1.12-1.17)
Excluding participants with baseline cardiovascular disease and hypertension	5202/138788	0.97 (0.94-1.00)	0.048	1.00 (0.96-1.03)	0.769	0.94 (0.90-0.99)	0.011	0.92 (0.87-0.96)	<0.001	1.11 (1.06-1.15)	<0.001	1.09 (1.04-1.14)
Not Adjusting for Occupation	14236/311892	0.94 (0.92-0.96)	<0.001	0.96 (0.95-0.98)	<0.001	0.92 (0.89-0.95)	<0.001	0.89 (0.87-0.92)	<0.001	1.17 (1.14-1.19)	<0.001	1.15 (1.13-1.18)
Not Adjusting for Family History of Diabetes	14236/311892	0.94 (0.93-0.96)	<0.001	0.97 (0.95-0.99)	<0.001	0.92 (0.89-0.94)	<0.001	0.89 (0.86-0.91)	<0.001	1.17 (1.14-1.19)	<0.001	1.16 (1.13-1.18)
Not Adjusting for Occupation and Family History of Diabetes	14236/311892	0.94 (0.93-0.96)		0.97 (0.95-0.99)	<0.001	0.91 (0.89-0.94)	<0.001	0.89 (0.86-0.91)	<0.001	1.17 (1.14-1.19)	<0.001	1.16 (1.13-1.18)
Excluding alcohol from UPF and PF variables	14236/311892	/	/	/	/	0.89 (0.86-0.93)	<0.001	0.82 (0.78-0.86)	<0.001	1.15 (1.13-1.18)	<0.001	1.14 (1.12-1.17)
Adjusting for Menopause and use of pill or hormone-replacement therapy (in females)	6657/184927	0.92 (0.89-0.95)		0.95 (0.92-0.98)	0.001	0.92 (0.87-0.97)	0.001	0.84 (0.80-0.89)	<0.001	1.17 (1.13-1.21)	<0.001	1.17 (1.13-1.21)

Nova group intakes expressed as a percentage of daily dietary intake (%g/day). Hazard ratios expressed per 10%g/day increase in Nova group intake. Cox proportional hazard models, with age as the underlying time variable. Time at entry was age at recruitment, and exit time was age at type 2 diabetes mellitus diagnosis, end of follow-up, loss to follow-up, or death, whichever came first. Model 2 was adjusted for sex, occupation, study centre, education level, smoking status and intensity, physical activity level, alcohol intake, family history of diabetes and total energy intake. Model 2 was further adjusted for saturated fat, sugar, sodium and Mediterranean Diet (model 5). Abbreviations: 95%CI: 95% confidence interval; MPF: unprocessed/minimally processed food; PCI: processed culinary ingredients; PF: processed food; SD: standard deviation; UPF: ultra-processed food.

Supplementary Table S13. The association between Nova group intake and incident type 2 diabetes mellitus with upper- and lower-bound scenario estimates, and with alternative Nova group metrics.

Cases/Numbers: 14236/311892	Mean (SD)	Model 2 HR (95%CI)	Model 2 p-value	Model 5 HR (95%CI)	Model 5 p-value
**Upper-bound scenario (%g/day) Per 10%g increment**					
MPF+PCI	69.2 (12.4)	0.94 (0.93-0.96)	<0.001	0.97 (0.95-0.98)	<0.001
PF	10.7 (9.0)	0.93 (0.90-0.96)	<0.001	0.94 (0.91-0.97)	<0.001
UPF	20.1 (8.9)	1.12 (1.10-1.15)	<0.001	1.08 (1.06-1.11)	<0.001
**Lower-bound scenario (%g/day) Per 10%g increment**					
MPF+PCI	74.3 (11.8)	0.93 (0.92-0.95)	<0.001	0.96 (0.94-0.98)	<0.001
PF	14.6 (9.8)	0.91 (0.89-0.94)	<0.001	0.89 (0.86-0.91)	<0.001
UPF	11.1 (7.5)	1.19 (1.16-1.21)	<0.001	1.18 (1.15-1.21)	<0.001
**Kcal/day per 100kcal increment**					
MPF+PCI	918.8 (339.6)	0.998 (0.990-1.005)	0.531	1.01 (1.00-1.02)	0.003
PF	580.6 (325.1)	0.973 (0.964-0.982)	<0.001	0.96 (0.95-0.97)	<0.001
UPF	682.3 (394.2)	1.023 (1.015-1.031)	<0.001	1.02 (1.01-1.03)	<0.001
**%kcal/day per 10%kcal increment**					
MPF+PCI	42.8 (12.5)	1.01 (0.99-1.03)	0.329	1.04 (1.03-1.06)	<0.001
PF	26.2 (11.3)	0.91 (0.89-0.93)	<0.001	0.89 (0.87-0.91)	<0.001
UPF	31.0 (14.4)	1.06 (1.04-1.08)	<0.001	1.04 (1.02-1.06)	<0.001
**g/day per 100g increment**					
MPF+PCI	2005.8 (824.4)	1.00 (1.00-1.01)	0.021	1.01 (1.00-1.01)	<0.001
PF	392.4 (322.4)	1.00 (0.99-1.01)	0.651	0.99 (0.98-1.00)	0.218
UPF	360.7 (261.8)	1.06 (1.05-1.07)	<0.001	1.07 (1.06-1.07)	<0.001

Nova group intakes expressed as a percentage of daily dietary intake (%g/day). Hazard ratios expressed per 10%g/day increase in Nova group intake. Cox proportional hazard models, with age as the underlying time variable. Time at entry was age at recruitment, and exit time was age at type 2 diabetes mellitus diagnosis, end of follow-up, loss to follow-up, or death, whichever came first. Model 2 was adjusted for sex, occupation, study centre, education level, smoking status and intensity, physical activity level, alcohol intake, family history of diabetes and total energy intake. Model 2 was further adjusted for saturated fat, sugar, sodium and Mediterranean Diet (model 5). Abbreviations: 95%CI: 95% confidence interval; MPF: unprocessed/minimally processed food; PCI: processed culinary ingredients; PF: processed food; SD: standard deviation; UPF: ultra-processed food.

Supplementary Table S14. Dietary sensitivity adjustments for the association between Nova group intake and incident type 2 diabetes mellitus.

Cases/Numbers: 14236/311892	MPF+PCI				PF				UPF			
**per 10%g/day increment**	**Model 2 HR (95%CI)**	**Model 2 p-value**	**Model 5 HR (95%CI)**	**Model 5 p-value**	**Model 2 HR (95%CI)**	**Model 2 p-value**	**Model 5 HR (95%CI)**	**Model 5 p-value**	**Model 2 HR (95%CI)**	**Model 2 p-value**	**Model 5 HR (95%CI)**	**Model 5 p-value**
Not Adjusting for total energy	0.94 (0.92-0.96)	<0.001	0.97 (0.95-0.99)	<0.001	0.94 (0.92-0.96)	<0.001	0.97 (0.95-0.99)	<0.001	1.16 (1.14-1.19)	<0.001	1.15 (1.13-1.18)	<0.001
Adjusting for EatWell Guide adherence	0.95 (0.93-0.97)	<0.001	0.96 (0.94-0.98)	0.0001	0.91 (0.89-0.94)	<0.001	0.89 (0.86-0.92)	<0.001	1.16 (1.14-1.19)	<0.001	1.16 (1.13-1.19)	<0.001
Adjusting for NutriScore	0.92 (0.91-0.94)	<0.001	0.94 (0.92-0.96)	<0.001	0.92 (0.89-0.95)	<0.001	0.90 (0.87-0.92)	<0.001	1.21 (1.18-1.24)	<0.001	1.20 (1.17-1.23)	<0.001
Adjusting for inflammatory score of the diet	0.95 (0.93-0.97)	<0.001	0.95 (0.93-0.97)	<0.001	0.90 (0.88-0.93)	<0.001	0.89 (0.86-0.92)	<0.001	1.17 (1.14-1.19)	<0.001	1.17 (1.14-1.20)	<0.001
Adjusting for fibre	0.96 (0.94-0.98)	<0.001	0.98 (0.96-1.00)	0.047	0.90 (0.88-0.93)	<0.001	0.87 (0.85-0.90)	<0.001	1.15 (1.12-1.17)	<0.001	1.14 (1.12-1.17)	<0.001
Adjusting for fruit and vegetables (not including juices)	0.94 (0.92-0.96)	<0.001	0.93 (0.91-0.95)	<0.001	0.91 (0.88-0.93)	<0.001	0.90 (0.88-0.93)	<0.001	1.17 (1.15-1.20)	<0.001	1.21 (1.18-1.24)	<0.001
Adjusting for protein, carbohydrate, sugar, total fat, saturated, monounsaturated and polyunsaturated fat, sodium, and fibre	0.93 (0.91-0.95)	<0.001	0.93 (0.92-0.95)	<0.001	0.89 (0.87-0.92)	<0.001	0.91 (0.88-0.94)	<0.001	1.20 (1.17-1.23)	<0.001	1.19 (1.16-1.22)	<0.001

Nova group intakes expressed as a percentage of daily dietary intake (%g/day). Hazard ratios expressed per 10%g/day increase in Nova group intake. Cox proportional hazard models, with age as the underlying time variable. Time at entry was age at recruitment, and exit time was age at type 2 diabetes mellitus diagnosis, end of follow-up, loss to follow-up, or death, whichever came first. Model 2 was adjusted for sex, occupation, study centre, education level, smoking status and intensity, physical activity level, alcohol intake, family history of diabetes and total energy intake. Model 2 was further adjusted for saturated fat, sugar, sodium and Mediterranean Diet (model 5). Abbreviations: 95%CI: 95% confidence interval; MPF: unprocessed/minimally processed food; PCI: processed culinary ingredients; PF: processed food; SD: standard deviation; UPF: ultra-processed food.

Supplementary Table S15. The association between MPF and PCI intake and incident type 2 diabetes mellitus as separate variables.

**MPF and PCI entered separately**<br>**Cases/Numbers: 14236/311892**	**Model 2 HR (95%CI)**	**Model 2 p-value**	**Model 5 HR (95%CI)**	**Model 5 p-value**
**per 10%g/day increment**				
MPF	0.95 (0.93-0.97)	<0.001	0.97 (0.96-0.99)	<0.007
PCI	0.18 (0.14-0.22)	<0.001	0.17 (0.14-0.22)	<0.001

Nova group intakes expressed as a percentage of daily dietary intake (%g/day). Hazard ratios expressed per 10%g/day increase in Nova group intake. Cox proportional hazard models, with age as the underlying time variable. Time at entry was age at recruitment, and exit time was age at type 2 diabetes mellitus diagnosis, end of follow-up, loss to follow-up, or death, whichever came first. Model 2 was adjusted for sex, occupation, study centre, education level, smoking status and intensity, physical activity level, alcohol intake, family history of diabetes and total energy intake. Model 2 was further adjusted for saturated fat, sugar, sodium and Mediterranean Diet (model 5). Abbreviations: 95%CI: 95% confidence interval; MPF: unprocessed/minimally processed food; PCI: processed culinary ingredients; PF: processed food; SD: standard deviation; UPF: ultra-processed food.

References

1. Monteiro CA, Cannon G, Moubarac JC, Levy RB, Louzada MLC, Jaime PC. The UN Decade of Nutrition, the NOVA food classification and the trouble with ultra-processing. Public Health Nutr. 2018 Jan;21(1):5–17.

2. Scheelbeek P, Green R, Papier K, Knuppel A, Alae-Carew C, Balkwill A, et al. Health impacts and environmental footprints of diets that meet the Eatwell Guide recommendations: analyses of multiple UK studies. BMJ Open. 2020 Aug 1;10(8):e037554.

3. Public Health England. Government recommendations for energy and nutrients for males and females aged 1–18 years and 19+ years [Internet]. 2016 [cited 2021 Oct 14]. Available from: https://assets.publishing.service.gov.uk/government/uploads/system/uploads/attachment_data/file/618167/government_dietary_recommendations.pdf

4. NHS. The Eatwell Guide [Internet]. [cited 2021 Oct 11]. Available from: https://www.nhs.uk/live-well/eat-well/the-eatwell-guide/
